# The dynamic nature of refugee children's resilience: a cohort study of Syrian refugees in Lebanon

**DOI:** 10.1017/S2045796022000191

**Published:** 2022-06-15

**Authors:** C. M. Popham, F. S. McEwen, E. Karam, J. Fayyad, G. Karam, D. Saab, P. Moghames, M. Pluess

**Affiliations:** 1 Department of Biological and Experimental Psychology, Queen Mary University of London, London, UK; 2 Institute for Development, Research, Advocacy and Applied Care, Beirut, Lebanon; 3 Saint George Hospital University Medical Center, Beirut, Lebanon; 4 Faculty of Medicine, Balamand University, El-Koura, Lebanon; 5 Medecins du Monde, Beirut, Lebanon

**Keywords:** Refugees, trauma, common mental disorders, social environment, children and adolescents

## Abstract

**Aims:**

Children's responses to war and displacement are varied; many struggle, while others appear resilient. However, research into these outcomes disproportionately focuses on cross-sectional data in high-income countries. We aimed to (1) investigate change in resilience across two timepoints in a highly vulnerable sample of Syrian refugee children in Lebanon, and (2) explore predictors of their mental health problems across time.

**Methods:**

In total, 982 Syrian child–caregiver dyads living in refugee settlements in Lebanon completed questionnaires via interview at baseline and follow-up one year later. We categorised children into groups based on their risk for mental health problems across both timepoints (stable high risk/SHR, deteriorating, improving, stable low risk) according to locally validated cut-offs on measures of post-traumatic stress disorder (PTSD), depression and behavioural problems. Analyses of covariance identified how the groups differed on a range of individual and socio-environmental predictors, followed up by cross-lagged panel models (CLPMs) to investigate the directionality of the relationships between significantly related predictors and symptoms.

**Results:**

The sample showed a meaningful amount of change in mental health symptoms from baseline to follow-up. Over half (56.3%) of children met SHR criteria and 10.3% deteriorated over time, but almost one-quarter (24.2%) showed meaningful improvement, and 9.2% were consistently at low risk for mental health problems at both timepoints. Several predictors differentiated the groups, particularly social measures. According to CLPMs, maternal acceptance (*β* = −0.07) predicted child mental health symptoms over time. Self-esteem (*β* = −0.08), maternal psychological control (*β* = 0.10), child maltreatment (*β* = 0.09) and caregiver depression (*β* = 0.08) predicted child symptoms and vice versa (*β*_se_ = −0.11, *β*_b_ = 0.07, *β*_mpc_ = 0.08, *β*_cm_ = 0.1, *β*_cd_ = 0.11). Finally, child symptoms predicted loneliness (*β* = 0.12), bullying (*β* = 0.07), perceived social support (*β* = −0.12), parent–child conflict (*β* = 0.13), caregiver PTSD (*β* = 0.07), caregiver anxiety (*β* = 0.08) and the perceived refugee environment (*β* = −0.09).

**Conclusions:**

Our results show risk and resilience are dynamic, and the family environment plays a key role in children's response to war and displacement. Conversely, children also have a significant impact on the family environment and caregiver's own mental health. Interventions to promote resilience in refugee children should therefore consider family-wide mechanisms.

There are 5.6 million Syrian refugees worldwide, half of whom are children. Most have been exposed to a wide range of war experiences, displacement and post-displacement adversities. Many resettle in unstable contexts such as informal settlements (United Nations High Commissioner for Refugees (UNHCR), 2020). Despite these challenges, children's mental health varies substantially; while many develop mental health problems including post-traumatic stress disorder (PTSD), depression, and behavioural problems (Kien *et al*., 2019; Blackmore *et al*., 2020; Henkelmann *et al*., 2020), a notable proportion show no evidence of such difficulties (Müller *et al*., 2019; Scherer *et al*., 2020). Given the extreme nature of the adversity refugee children face, we argue these children demonstrate manifested resilience, defined as better than expected development in the context of adversity (Masten, 2016; Miller-Graff, 2020).

Better understanding of refugee children's resilience could inform interventions for those struggling, but definitions of resilience vary (Cosco *et al*., 2017). While some define resilience based on available resources, others focus on developmental outcomes of a putative process of resilience (i.e. manifested resilience; Miller-Graff 2020). However, the process of adapting to adversity can take different trajectories (Popham *et al*., 2021). Children struggling at one timepoint may recover, while continuing accumulation of stressors may cause a child originally doing well to deteriorate (Müller *et al*., 2019). This could be particularly complex in populations exposed to ongoing adversity, such as refugees living in camps. Research thus far suggests that the mental health of conflict-affected children generally improves over time, but some children may not improve, and some may deteriorate (Müller *et al*., 2019; Hermosilla *et al*., 2021).

Many individual and socio-environmental factors, such as coping strategies or social support, have been linked to refugee child mental health, but much of this research comes from high-income countries and cross-sectional data (Scharpf *et al*., 2021). Longitudinal research to date emphasises the importance of the family environment: caregiver mental health, parenting, and other aspects of family functioning are predictive of emotional and behavioural problems in refugee children (Panter-Brick *et al*., 2014; Sangalang *et al*., 2017; Bryant *et al*., 2018). However, although the focus is often on how socio-environmental factors impact the child, Syrian refugee mothers report how their children's mental health can also affect their own mental health and parenting (Rizkalla *et al*., 2020). Further longitudinal research is needed to investigate such reciprocal relationships between children and their environment.

We aimed to further the research on child resilience following war and displacement, using two waves of data from Syrian refugee children included in the BIOPATH study (McEwen *et al*., 2022*b*). Specifically, we had three key aims: (1) identify the proportion of children at low risk for mental health problems in our sample and describe changes over time; (2) identify predictors of change in risk and resilience; (3) investigate the directionality of the relationships between identified predictors and mental health symptoms over time. We used low risk for clinical levels of PTSD, depression and externalising to approximate manifested resilience.

## Methods

### Study design

We addressed our aims using two waves of data from a large sample of Syrian refugee child–caregiver dyads from the BIOPATH cohort study (McEwen *et al*., 2022*b*). First, we created four groups based on change in risk for three common mental health problems in response to war and displacement (Kien *et al*., 2019) from baseline to follow-up: (1) children with low symptoms on PTSD, depression and externalising behaviour problems at both waves (stable low risk/SLR), (2) children with low symptom scores on all three outcomes at baseline whose symptoms meaningfully worsened at follow-up (deteriorating), (3) children with high symptoms at baseline who showed meaningful improvement at follow-up (improving) and (4) children with continuously high symptom scores on any outcomes at both waves (stable high risk/SHR). We ran group comparisons to determine what factors characterised each of the four groups, and finally investigated the directionality of associations between children's mental health symptoms and the predictors identified in group comparisons using cross-lagged panel models (CLPMs). All procedures contributing to this work comply with the ethical standards of the relevant national and institutional committees on human experimentation (McEwen *et al*., 2022*b*).

### Setting and participants

Data were collected in the Beqaa region of Lebanon in 2017–2019. We used purposive cluster sampling, approaching small-to-medium-sized ITSs representing a range of vulnerabilities according to the UNHCR vulnerability index (McEwen *et al*., 2022*b*). Following agreement with community leaders, we approached all families present, and invited one child per eligible family (i.e. child aged 8–16 years, left Syria in the preceding 4 years, primary caregiver available) to participate. If more than one child in a family was eligible we invited the child whose birthday was closest to the recruitment date, to avoid selection bias. Informed consent and assent were given by each caregiver and child, respectively. Questionnaire data were collected by a team of interviewers in the settlements. Interviews took approximately 50–60 min. All measures were repeated one year later with approximately two-thirds of the original baseline sample. For a more detailed explanation of recruitment, see McEwen *et al*. (2022*b*).

### Variables

All participants were interviewed in their homes by trained (online Supplementary 1.1), local, native Arabic-speaking interviewers. Different interviewers conducted the child and caregiver interviews simultaneously. Some measures were exclusively child or caregiver reported, while others were reported by both (online Supplementary Table S1).

#### Mental health outcomes

The primary outcomes were self-reported PTSD (Child PTSD Symptom Scale/CPSS, Foa *et al*., 2001), self-reported depression (Centre for Epidemiological Studies Depression Scale for Children/CES-DC, abridged, Faulstich *et al*., 1986), and parent-reported externalising behaviour problems, measured using the externalising subscale of the Strengths and Difficulties Questionnaire (SDQ, Goodman, 1997) and additional items related to conduct disorder and oppositional defiant disorder administered separately (McEwen *et al*., 2022*b*). Scales were chosen according to availability of Arabic versions and validity in similar populations. Following pilot testing with Syrian refugees in Lebanon, the CES-DC was abridged to ten items and minor changes to phrasing (including Arabic dialect) were made to the CES-DC and CPSS (McEwen *et al*., 2020; online Supplementary 1.1). Cut-off scores on each outcome (12 out of 51 on the adjusted CPSS, 10 out of 30 on the adjusted CES-DC, and 12 out of 44 on the combined externalising scale total) were derived from structured clinical interviews (MINI-KID, Sheehan *et al*., 2010) and clinical judgement in a representative subsample (*n* = 119) of the cohort (McEwen *et al*., 2020). Cut-offs had sensitivity of 81–85%, but specificity fell below 80%, meaning that some children flagged as at risk may not represent clinical cases. Children below cut-offs likely do not have clinical symptoms (negative predictive value of 79–91%). For more detailed information see online Supplementary 1.2. Finally, we measured wellbeing using the World Health Organisation – Five Wellbeing Index (Bech, 2012; Topp *et al*., 2015).

#### Predictor variables

*Individual and social factors*: We investigated a variety of individual and social predictors that have been associated with children's mental health in previous research (online Supplementary Table S1). Individual-level predictors included optimism (Ey *et al*., 2005), self-efficacy (Schwarzer and Jerusalem, 1995), a single self-esteem item (Harris *et al*., 2018), the temperament trait of environmental sensitivity (Pluess *et al*., 2018), coping strategies (Program for Prevention Research, 1999), future orientation (McEwen *et al*., 2022*b*), and a single item on the child's general health (McEwen *et al*., 2022*b*). The social environment measures included aspects of the caregiver–child relationship (maternal acceptance, Schaefer, 1965; parental monitoring, Barber, 1996; parent–child conflict, Barber 1999; child maltreatment, Runyan *et al*., 2009; maternal psychological control, Barber *et al*., 2012; positive home experiences, McEwen *et al*., 2022*b*), the caregiver's own mental and general health (depression, Radloff, 1977; anxiety, Henry and Crawford, 2005; PTSD, Blevins *et al*., 2015; a single general health item, McEwen *et al*., 2022*b*), relationships within and beyond the family (loneliness, Asher *et al*., 1984; perceived social support, Ramaswamy *et al*., 2009; bullying, McEwen *et al*., 2022*b*), and the child's home and employment responsibilities (McEwen *et al*., 2022*b*). Finally, caregivers reported their literacy, income, employment status, household size, and aspects of the wider environment (collective efficacy, Sampson *et al*., 1997; human insecurity, Ziadni *et al*., 2011; perceived refugee environment, McEwen *et al*., 2022*b*). For detailed information, see online Supplementary Table S1.

*Exposure to war*: War exposure was measured with the *War Events Questionnaire* (WEQ), a 25-item checklist of war events reported at baseline (Karam *et al*., 1999). In line with recommendations for multiple informant approaches to war exposure (Oh *et al*., 2018), child and caregiver responses were combined such that if either one reported that the child experienced an event, the event was considered to have occurred.

### Statistical methods

Analyses were conducted in RStudio. Multiple imputation using Fully Conditional Specification in the mice package (van Buuren and Groothuis-Oudshoorn, 2011) was applied to impute the small number of missing data. We imputed all missing measures for the analysis, bar demographic variables, war exposure, and child mental health. We ran all analyses in both the imputed (*N* = 982) and original (*N* = 861) datasets and report the pooled imputation estimates in the main text of this paper. Complete case analyses are reported in online Supplementary sections 2.4 and 2.5.

#### Aim 1: change in risk and resilience

In order to investigate risk and resilience over time, we calculated the frequencies of four basic groups of mental health risk (SHR, deteriorating, improving, and SLR) using a two-step approach. At each wave, we created high- and low-risk groups using the locally validated clinical cut-offs for PTSD, depression, and externalising problems. If participants scored above the cut-off for *any* of the three measures, they were classed as in the high-risk group but if participants scored below *all three* cut-offs, they were classed in the low-risk group (i.e. resilience). We then adjusted the groupings at follow-up according to which children showed *meaningful* change at follow-up, defined as crossing the relevant cut-off(s) from baseline to follow-up to meet the low or high-risk criteria (i.e. below all cut-offs *v*. above any) paired with a change in symptom score of at least 20% on the relevant scale. Children who did not show meaningful change were classed as SHR/SLR. This ensured that small amounts of variability in reporting over time were not counted as categorical change.

#### Aim 2: group characteristics

Specific characteristics of the four groups were identified with a series of individual analyses of covariance for each predictor to compare their baseline scores and the change over time from baseline to follow-up. For each predictor we considered the effect of group membership on the baseline score controlling for change score, then on the change over time while controlling for the baseline score. In each model we also controlled for the effects of war exposure, age, gender, and time since leaving Syria. The significance level of each model was corrected using the Benjamini–Hochberg correction to account for the total number of models tested (Benjamini and Hochberg, 1995).

#### Aim 3: directionality of predictor – mental health relationships

Each predictor that was significantly associated with group differences in Aim 2 was further investigated using CLPMs in order to investigate the directionality of effect. However, in place of the categorical grouping, we used a continuous mental health symptom composite score to improve power. This was calculated by taking the average of the three primary outcome measures (PTSD, depression, and externalising) each adjusted for the number of items per scale. We ran a series of CLPMs using the semTools package (Jorgensen *et al*., 2021) containing the child mental health symptom composite at both waves, and the predictor of interest (e.g. self-esteem) at both waves. The models included autoregressive and cross-lagged paths, and within-time covariance. As with Aim 2, we controlled for the effects of age, gender, time since leaving Syria, and war exposure on baseline and follow-up scores for the predictor variable and symptom score (online Supplementary Fig. S1 illustrates the model format).

## Results

### Descriptive data

The final sample consisted of 982 child–caregiver dyads with data at both waves (Table 1). Approximately half (52.9%) the children were female, and at baseline children's average age was 11.22 years (s.d. = 2.34), 42.4% had left Syria in the past 3 years, and the remainder had left more than 3 years previously. Children reportedly experienced up to 24 (*M* = 9.57, s.d. = 5.47) different types of war events. The majority (91.1%) of participating caregivers were the child's mother. The proportion of children above clinical cut-offs at baseline and follow-up was 54.9 and 34.4% respectively for PTSD, 37.8 and 27% for depression, and 43.9 and 41.9% for externalising behaviour problems. This longitudinal sample represented 61.7% of the baseline sample, and showed no differences likely to create substantial bias (online Supplementary 2.1; McEwen *et al*., 2022*b*).

**Table 1.** Sample characteristics

	Whole sample (*N* = 982)	Stable high risk (SHR) (*n* = 553)	Deteriorating (D) (*n* = 101)	Improving (I) (*n* = 238)	Stable low risk (SLR) (*n* = 90)	*F*/*χ*^2^	Likelihood/post-hoc comparisons
Child age, mean (s.d.)	11.22 (2.34)	11.3 (2.39)	10.75 (2.22)	11.29 (2.40)	11.06 (1.90)	1.76	
Child gender, % female	52.9	49.5	51.5	55.5	67.8	11.19*	Girls more likely to be SLR
Caregiver relationship to child, % mother	91.1	90.6	95.0	90.8	91.1	2.16	
Time since leaving Syria, % ⩽3 years	42.4	43.8	36.6	42.4	40.0	2.01	
Child war exposure, mean (s.d.)	9.57 (5.47)	10.47 (5.55)	7.54 (4.98)	8.98 (5.24)	7.88 (4.96)	13.87***	D/I/SLR < SHR
Wave 1 child symptoms, mean (s.d.)^a^	0.28 (0.14)	0.33 (0.13)	0.12 (0.05)	0.29 (0.11)	0.10 (0.04)	172.72***	SLR/D < I < SHR
Wave 2 child symptoms, mean (s.d.)^a^	0.22 (0.15)	0.30 (0.14)	0.26 (0.12)	0.09 (0.05)	0.09 (0.06)	245.93***	SLR/I < D < SHR

*Note*: Descriptive statistics on key demographics and change in mental health. Analyses of variance/*χ*^2^ tests used where appropriate to compare the groups, and Tukey's post-hoc tests reported for significant ANOVAs. *p < 0.05, **p < 0.01, ***p < 0.001.

a Child mental health symptom composite score (average of PTSD, depression and externalising symptom scores, adjusted for number of items).

#### Aim 1: change in risk and resilience

**Fig. 1.**
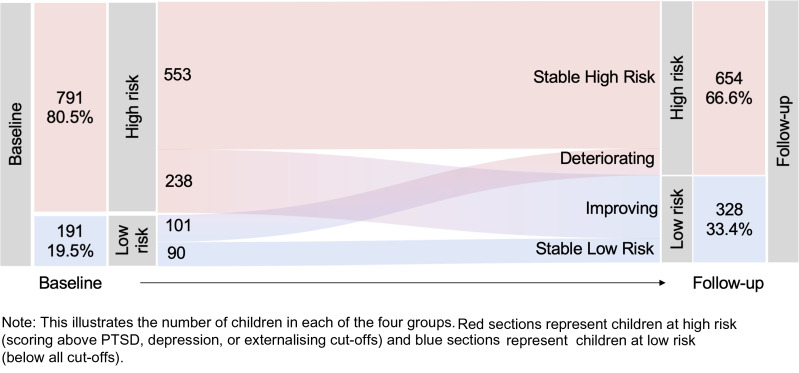
Mental health risk change from baseline to follow-up.

The percentage of children meeting the low-risk criteria increased from 19.5% at baseline to 33.4% at follow-up, but all four groups (SHR, deteriorating, improving, SLR) were represented in the data (Fig. 1). Of the originally 791 high-risk cases at baseline, 553 (69.9%) remained in the high-risk group (scoring above at least one cut-off) at follow-up (SHR), while 238 (30.1%) moved below all cut-offs, showing a reduction in symptoms of at least 20% (*M* = 65.0%) on the relevant outcomes (improving). Of the 191 children with low risk at baseline, 90 (47.1%) remained below all cut-offs at follow-up (SLR) whilst 101 (52.9%) showed increased risk and scored above at least one cut-off at follow-up (deteriorating), with an increase in symptoms of at least 20% (*M* = 166.7%). At baseline and follow-up, children meeting low-risk criteria reported significantly higher wellbeing (*M*_w1_ = 74.79, s.d._w1_ = 19.44; *M*_w2_ = 78.12, s.d._w2_ = 18.85) compared to those meeting high-risk criteria (*M*_w1_ = 65.47, s.d._w1_ = 26.95; *t*_w1_(387.19) = −5.47, *p*_w1_ < 0.001, *d*_w1_ = 0.4; *M*_w2_ = 66.61, s.d._w2_ = 29.39; *t*_w2_(918.16) = −7.41, *p*_w2_ < 0.001, *d*_w2_ = 0.47). SHR children had significantly higher reported war exposure (*M* = 10.47) compared to all other groups (*M*_D_ = 7.54; *M*_I_ = 8.98; *M*_SLR_ = 7.88; *F*_(3, 978)_ = 13.87, *p* < 0.001). Girls were more likely to be SLR (*χ*^2^ = 11.19, *p* = 0.011). See Table 1 for further group comparisons.

#### Aim 2: group characteristics

The four groups differed significantly on a range of variables at baseline and in change over time (Table 2). The improving group was characterised by better perceived refugee environment at baseline compared to the other groups. The SHR group differed from the other groups on a larger number of variables, characterised by lower baseline scores on several protective/promotive factors, higher baseline scores on a range of social risk factors, and greater increases in loneliness and social isolation and maternal psychological control over time. Change in a range of factors significantly differentiated children with low risk (improving and SLR risk groups) from those with higher risk (deteriorating and SHR risk groups) at follow-up (Table 2).

**Table 2.** Analyses of covariance: results from significant models

Factor		Mental health risk group *M* (s.d.)				*F*	Adj. *R*^2^	Post-hoc comparisons
		Stable high risk (SHR)	Deteriorating (D)	Improving (I)	Stable low risk (SLR)			
Optimism	Baseline	8.82 (3.14)	9.65 (2.51)	9.5 (2.73)	9.87 (2.43)	9.71***	0.42	SHR < D/I/SLR
	Change	0.45 (4.21)	0.2 (3.93)	0.71 (3.66)	0.67 (3.27)	6.03**	0.40	SHR < I/SLR
Self-esteem	Baseline	3.88 (1.27)	4.41 (0.64)	4.13 (1.04)	4.44 (0.69)	10.76***	0.47	SHR < D/I/SLR
	Change	0.1 (1.41)	−0.3 (1.04)	0.21 (1.15)	−0.07 (0.99)	5.86**	0.41	SHR/D < I/SLR
Environmental sensitivity	Baseline	5.1 (1.01)	4.76 (0.98)	5.07 (1.05)	4.87 (0.93)	6.78***	0.52	I/SLR < SHR
	Change	−0.23 (1.34)	0.16 (1.31)	−0.56 (1.24)	−0.26 (1.21)	10.97***	0.50	I/SLR < SHR/D
Future planning	Baseline	3.03 (0.9)	3.05 (0.78)	3.01 (0.88)	2.94 (0.95)	2.01		
	Change	0.05 (1.19)	−0.08 (1.11)	−0.22 (1.22)	0.03 (1.19)	4.95**	0.40	I < SHR
Distraction coping	Baseline	6.45 (2.3)	6.45 (2.19)	6.45 (2.29)	6.38 (1.88)	2.78		
	Change	−0.71 (3.16)	−1.16 (2.71)	−0.26 (3.11)	−0.06 (2.59)	6.20**	0.42	SHR/D < I/SLR
Child general health^a^	Baseline	2.37 (0.82)	2.13 (0.91)	2.28 (0.79)	2.12 (0.73)	7.00***	0.47	I/SLR < SHR
	Change	−0.31 (1.01)	−0.08 (1.19)	−0.48 (0.94)	−0.35 (0.92)	7.45***	0.46	I/SLR <SHR/D
Bullying	Baseline	5.7 (6.83)	3.18 (5.31)	3.98 (5.79)	2.78 (4.54)	12.52***	0.50	D/I/SLR < SHR
	Change	−1.23 (7.93)	0.58 (6.68)	−1.94 (7.09)	−0.82 (5.55)	8.03***	0.45	I < SHR/D SLR < SHR
Loneliness and social isolation	Baseline	8.72 (3.02)	7.02 (2.37)	8.23 (2.69)	7.01 (2.49)	23.77***	0.50	D < SHR SLR < I < SHR
	Change	−0.96 (4.12)	−0.19 (4.05)	−1.61 (3.78)	−0.88 (3.39)	12.65***	0.47	I < SHR SLR < D < SHR
Perceived social support	Baseline	5.5 (0.97)	5.68 (0.8)	5.61 (0.81)	5.72 (0.76)	3.27*	0.37	SHR < D/I/SLR
	Change	0.13 (1.31)	0.22 (1.2)	0.19 (1.05)	0.13 (1.03)	2.40		
Maternal acceptance	Baseline	26.79 (4.4)	27.82 (3.18)	27.41 (3.66)	28.28 (2.61)	7.90***	0.34	SHR < D/I/SLR
	Change	0.24 (5.39)	−0.04 (4.09)	0.63 (4.54)	0.52 (3.54)	3.69*	0.32	SHR < I/SLR
Maternal psychological control	Baseline	11.61 (2.86)	10.52 (1.5)	10.86 (2.05)	10.3 (1.65)	14.96***	0.49	D/I/SLR < SHR
	Change	−0.66 (3.29)	−0.31 (1.98)	−0.6 (2.21)	−0.13 (2.23)	4.68**	0.47	SHR < D/I/SLR
Parent–child conflict	Baseline	6.12 (3.25)	5.74 (2.97)	5.93 (3.29)	5.06 (2.33)	8.23***	0.37	SLR/I < SHR SLR < D
	Change	1.72 (4.97)	1.34 (4.53)	0.53 (4.52)	1.09 (3.8)	9.10***	0.38	I/SLR < SHR
Child maltreatment	Baseline	13.82 (13.52)	7.46 (8.27)	11.08 (11.81)	7.23 (8.21)	20.30***	0.50	I < SHR SLR < D < SHR
	Change	−3.09 (15.1)	1.41 (10.84)	−5.99 (13.63)	−3.3 (10.11)	17.13***	0.48	I/SLR < SHR/D
Caregiver depression	Baseline	16.19 (6.35)	12.44 (6.33)	15.2 (6.33)	13.12 (6.43)	24.1***	0.36	SLR < I/D < SHR
	Change	−0.31 (7.87)	1.46 (7.71)	−4.52 (8.64)	−3.79 (8.26)	37.93***	0.36	I/SLR < SHR/D
Caregiver PTSD	Baseline	35.95 (17.33)	27.64 (14.62)	34.93 (17.9)	29.01 (18.25)	14.12***	0.47	SLR < D < SHR I < SHR
	Change	−8.08 (22.39)	−2.42 (20.7)	−17.75 (25.19)	−13.04 (21.22)	23.82***	0.45	I/SLR < SHR/D
Caregiver anxiety	Baseline	8.69 (5.39)	6.25 (4.83)	8.36 (5.34)	7.28 (5.36)	9.35***	0.43	D/I/SLR < SHR
	Change	−0.88 (6.58)	0.13 (5.72)	−3.08 (6.56)	−2.34 (6.89)	14.30***	0.41	I/SLR < SHR/D
Caregiver general health^a^	Baseline	3.04 (0.9)	2.84 (0.97)	2.97 (0.92)	2.8 (0.93)	4.12*	0.37	I/SLR < SHR
	Change	−0.18 (1.06)	−0.07 (1.07)	−0.41 (1.06)	−0.09 (1.13)	6.00**	0.37	I < SHR/D SLR < I
Human insecurity	Baseline	3.7 (0.37)	3.64 (0.41)	3.67 (0.43)	3.7 (0.42)	5.08**	0.51	I < SHR
	Change	0.08 (0.47)	0.15 (0.51)	−0.02 (0.61)	−0.03 (0.6)	7.71***	0.51	I/SLR < SHR/D
Perceived refugee environment	Baseline	3.21 (0.51)	3.27 (0.53)	3.28 (0.5)	3.14 (0.51)	9.39***	0.46	SHR/D/SLR < I
	Change	0.03 (0.6)	0.04 (0.61)	0.21 (0.61)	0.29 (0.63)	15.64***	0.42	SHR/D < I/SLR
Child responsibilities	Baseline	4.41 (3.49)	3.62 (2.81)	4.22 (3.28)	4.18 (2.75)	1.97		
	Change	1.08 (4.09)	1.76 (3.91)	0.47 (4.11)	1.2 (4.06)	4.48**	0.36	I < SHR/D

*Note*: Table representing descriptive statistics and analyses of covariance (ANCOVAs) from significant predictors using imputed data (*N* = 982). Child age, gender, time since leaving Syria and war exposure were entered as covariates into all ANCOVAs. Baseline models controlled for change scores, and change models controlled for baseline scores. *F* statistic is based on test against null model including only covariates. Adjusted *R*^2^ is based on full model. Post-hoc comparisons are based on Tukey's test. Means and s.d.s are unadjusted estimates, all other statistics are based on adjusted means according to the ANCOVA models. See online Supplementary Table S2 for all ANCOVA results. *p* Values based on Benjamini–Hochberg correction for multiple testing (Benjamini and Hochberg, 1995). *p < 0.05, **p < 0.01, ***p < 0.001.

a Higher scores on child and caregiver general health indicate worse health.

#### Aim 3: directionality of predictor – mental health relationships

**Fig. 2.**
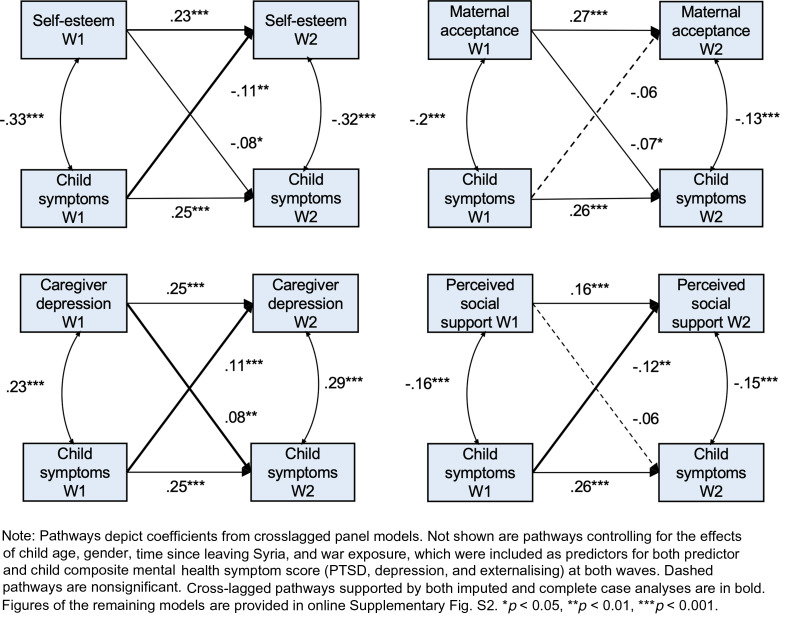
Cross-lagged panel models representing key predictors from the individual, family, and community systems.

For every predictor whose baseline or change score significantly differed between groups, CLPMs were used to investigate the direction of relationship between the predictor in question and the composite mental health symptom score. All CLPMs were just identified so there was no information about fit. Several cross-lagged pathways emerged as significant (Table 3). Some pathways were not significant in the complete case analysis due to reduced power (online Supplementary Fig. S3), so we report the imputed estimates for pathways that were significant in the imputed data and supported by similar trend-level estimates in the complete case data. Following those criteria, the key results were as follows. Baseline maternal acceptance (*β* = −0.07, *p* = 0.046) was predictive of later child mental health symptoms. Caregiver depression at baseline was predictive of child mental health symptoms at follow-up (*β* = 0.08, *p* = 0.009) and vice versa (*β* = 0.11, *p* < 0.001), as was the case for maternal psychological control (*β*_pc-mh_ = 0.10, *p*_pc-mh_ = 0.003; *β*_mh-pc_ = 0.08, *p*_mh-pc_ = 0.011), child maltreatment (*β*_m-mh_ = 0.09, *p*_m-mh_ = 0.009; *β*_mh-m_ = 0.1, *p*_mh-m_ = 0.005), and self-esteem (*β*_se-mh_ = −0.08, *p*_se-mh_ = 0.033; *β*_mh-se_ = −0.11, *p*_mh-se_ = 0.003). Baseline child mental health symptoms were predictive of optimism, loneliness and social isolation, bullying, perceived social support, parent–child conflict, caregiver PTSD, caregiver anxiety, and the perceived refugee environment at follow-up, but none of these predictors significantly predicted child symptoms at follow-up (online Supplementary Fig. S2). Figure 2 contains examples of key predictors from the individual, family, and wider systems that showed uni- and bi-directional cross-lagged relationships with child symptoms.

**Table 3.** Summary of cross-lagged panel models with significant cross-lagged pathways

	Child symptom auto-regressed pathway (a)	Predictor auto-regressed pathway (b)	W1 covariance (c)	W2 covariance (d)	Cross-lagged pathways	
					W1 predictor → W2 symptoms (e)	W1 symptoms → W2 predictor (f)
Optimism	0.26***	0.12**	−0.27***	−0.28***	−0.04	−0.08*
Self-esteem	0.245***	0.23***	−0.33***	−0.32***	−0.08* (−0.07)	−0.11**
Bullying	0.25***	0.2***	0.25***	0.28***	0.07* (0.06)	0.07* (0.08)
Loneliness and social isolation	0.26***	0.02	0.33***	0.39***	0.05	0.12**
Perceived social support	0.26***	0.16***	−0.16***	−0.15***	−0.06	−0.12**
Maternal acceptance	0.26***	0.27***	−0.17***	−0.13***	−0.07* (−0.07)	−0.06
Maternal psychological control	0.25***	0.24***	0.26***	0.20***	0.10**	0.08* (0.06)
Parent–child conflict	0.27***	0.07*	0.18***	0.21***	0.03	0.13***
Child maltreatment	0.24***	0.23***	0.37***	0.33***	0.09** (0.07)	0.1**
Caregiver depression	0.25***	0.25***	0.23***	0.29***	0.08**	0.11***
Caregiver PTSD	0.27***	0.10**	0.18***	0.26***	0.02	0.07*
Caregiver anxiety	0.27***	0.20***	0.18***	0.21***	−0.01	0.08*
PREI	0.27***	0.23***	−0.01	−0.22***	0.00	−0.09**

*Note*: Table depicting the coefficients and *p* values of the pathways from the cross-lagged panel models with significant cross-lagged pathways between predictor and child symptoms in either direction. Complete case estimates are shown in brackets where they differ from the imputed estimates. Letters a, b, c, d, e, f correspond to the pathway labels in online Supplementary Fig. S1: a = child symptom auto-regressed pathway; b = predictor auto-regressed pathway; c = W1 covariance; d = W2 covariance; e = cross lagged pathway: predictor → symptoms; f = cross lagged pathway: symptoms → predictor. *p < 0.05, **p < 0.01, ***p < 0.001.

## Discussion

Our aim was to investigate change in and predictors of risk and resilience over time in a sample of Syrian refugee children living in a particularly challenging context in Lebanon. The children were categorised into four groups based on their change in risk for mental health problems across two timepoints one year apart: SHR, deteriorating, improving, and SLR. Many predictors differentiated these groups from one another, but social and familial predictors were of particular importance, and showed reciprocal relationships with children's symptoms.

### Change in risk and resilience over time

Mental health in our sample was dynamic, and overall improved; a greater proportion of children met low-risk criteria at follow-up compared to baseline. In total, 9.2% of the sample were low risk at both waves (SLR) and 24.2% improved from showing likely clinical levels of PTSD, depression, and/or externalising behaviour problems at baseline to scoring below all three cut-offs at follow-up (improving). We used this as an indicator of resilience; children that met the improving and SLR criteria demonstrated evidence of resistance to or recovery from the psychological impact of war and displacement, and can therefore be described as resilient (Masten, 2016).

These findings fit with recent research finding overall improvements over time in children affected by conflict (Müller *et al*., 2019; Purgato *et al*., 2020; Hermosilla *et al*., 2021) and show that even in challenging post-displacement contexts some children demonstrate manifested resilience. However, 10.3% of the sample deteriorated over time, the proportion of children meeting externalising criteria remained notably higher than in previous reviews (Kien *et al*., 2019; Blackmore *et al*., 2020), and the proportion of children with persistently high general risk was larger than seen in children resettled in Europe (Müller *et al*., 2019). This emphasises the need for longitudinal research in a variety of contexts, as children doing relatively well across multiple measures can begin to struggle, and those doing poorly can improve. The key question is what helps or hinders the resilience process.

### Predictors of mental health and resilience

Results from our second and third aims provided some answers to this question. Predictors at the individual, family, and community levels were associated with children's mental health at baseline and over time, but CLPMs showed mental health at follow-up was primarily predicted by aspects of the immediate family context. At the individual level, self-esteem was associated with general low symptoms. Within the family, baseline maternal acceptance was predictive of later low child symptoms, while maternal psychological control, child maltreatment, and caregiver depression showed reciprocal relationships with child symptoms, and child symptoms were predictive of but not predicted by parent–child conflict and caregiver anxiety and PTSD. Child symptoms were also predictive of several factors in the wider social environment, including loneliness and perceived social support.

These results indicate some predictors that may be causally related to child mental health, in line with previous findings (Panter-Brick *et al*., 2014; Sangalang *et al*., 2017; Bryant *et al*., 2018), but also reflect a more complex story. The bidirectional relationships between children's symptoms and social factors demonstrate a vicious cycle of risk. For example, emotional or behavioural problems could significantly impact caregiver mental health and the parent–child relationship, resulting in increases in harsh parenting which in turn negatively affect the child. This accords with personal accounts from Syrian mothers, who report that both their children's and their own mental health impacts their parenting (Rizkalla *et al*., 2020). Beyond the family, noticeable symptoms may also impact social support due to peer stigmatisation (O'Driscoll *et al*., 2012) which could explain why child symptoms predict higher bullying and loneliness scores, and lower symptoms are associated with better social support. Treatment of child symptoms could therefore improve access to social resources.

However, the finding that caregiver depression and aspects of the parent–child relationship are predictive of later child symptoms emphasises the importance of a family-wide approach to treatment. Parenting interventions may be helpful for some families, but previous research suggests that parenting is also influenced by caregivers' own trauma and psychological distress (Sangalang *et al*., 2017; Bryant *et al*., 2018). There are multiple possible stressors in the refugee context, such as poor housing or food insecurity, which could additionally impact caregivers', and therefore children's, mental health (Li *et al*., 2016). In fact, we observed that improving children reported better baseline refugee environment scores than others. Psychological support for caregivers or systemic family therapy could bolster resources within the family, but practical and community-level support may provide a baseline from which other interventions are more effective in the longer term. Future research should explore the impact of the wider environment through the family system and to the child.

### Strengths and limitations

We provide novel findings looking at changes in risk and resilience over two waves of data, and the directionality of predictors of refugee mental health. This study is characterised by a longitudinal, challenging to reach sample that is representative of a large proportion of the global refugee population, the majority of whom reside in low- and middle-income countries, and, since 2014, have originated from Syria (UNHCR, 2020).

Despite these strengths, our methods had some limitations. First, we measured mental health using self-reported symptom scales. However, scales were extensively piloted and, where possible, modified to be context-appropriate. Furthermore, we derived cut-offs through clinical assessment in a subsample, choosing cut-offs with the best balance of sensitivity and specificity for our particular sample (McEwen *et al*., 2020). However, specificity fell below 80%, and consequently the high-risk groups may contain some false positives. Prevalence estimates adjusted for false positives and negatives are therefore lower than reported here (McEwen *et al*., 2022*a*), but adjustments cannot be applied at the individual level, so we retain unadjusted estimates. Secondly, PTSD, depression, and externalising problems may be differentially associated with some of the factors measured. However, we used the composite symptom score to complement our categorical approach, identify potential resilience factors, and identify associations between a child's general symptomatology and their environment (Jongedijk *et al*., 2020). Finally, a selection bias in recruitment and retention at follow-up cannot be excluded due to restricted access to certain settlements, reliance on presence of families during recruitment, and the high mobility of our sample. However, differences between the baseline and follow-up samples were small (McEwen *et al*., 2022*b*), meaning any substantial retention bias is unlikely.

## Conclusion

In our longitudinal analysis of Syrian refugee children in Lebanon, many showed meaningful changes in risk and resilience from baseline to one year later. The overall proportion of children with no evidence of clinical symptoms of PTSD, depression, or externalising behaviour problems, from which we can infer demonstration of resilience, increased over time, although approximately half of the originally low-risk children deteriorated from one year to the next. Our results agree with previous research on the importance of specific social and familial factors (Sangalang *et al*., 2017; Bryant *et al*., 2018; Scharpf *et al*., 2021) for risk and resilience but also provide evidence of directionality over time. In particular, findings indicate reciprocal relationships between children and caregiver's mental health, and aspects of the parent–child relationship, and identify ways in which child mental health impacts the social environment. Our results are most useful when considered in the context of environmental challenges that refugee families face, and their agency in the face of that challenge. Based on the results of our study, family-focused systemic psychosocial support may be a useful route to promoting resilience. However, more longitudinal research is needed to better understand the impact of the refugee environment on children.

## Data Availability

Researchers interested in accessing data should contact Professor Michael Pluess at Queen Mary University of London, UK (e-mail: m.pluess@qmul.ac.uk).

## Supplementary material

For supplementary material accompanying this paper visit http://doi.org/ 10.1017/S2045796022000191. click here to view supplementary material
