# Supplementary material for: The dynamic nature of refugee children's resilience: a cohort study of Syrian refugees in Lebanon
Source: Epidemiol Psychiatr Sci. 2022 Jun 15;31:e41. doi: 10.1017/S2045796022000191 (PMC9228593; doi:10.1017/S2045796022000191)
Supplement: Supplementary file 1 [file S2045796022000191sup001.pdf]

Supplementary Materials

The Dynamic Nature of Refugee Children's Resilience: A Cohort Study of Syrian Refugees in Lebanon

1. Supplemental Methods 2

  1.1 Piloting of Measures 2

  1.2 Mental Health Outcome Cut-Off Scores 2

  1.3 Table S1. Details of Measures 4

  1.4 Figure S1. Cross-Lagged Panel Models: Full Model Illustration 13

2. Supplemental Results 14

  2.1 Comparison of Current Sample to Baseline 14

  2.2 Table S2. Analyses of Covariance: Main Results from all Models 15

  2.3 Figure S2. Cross-Lagged Panel Models: Main Results 21

  2.4 Table S3. Analyses of Covariance: Complete Case Results 26

  2.5 Figure S3. Cross-Lagged Panel Models: Complete case data 32

3. References 37

1. Supplemental Methods

1.1 Piloting of Measures

Where an Arabic translation was not available, measures were translated using a standard protocol (McEwen *et al.* 2022). Two local clinical psychology students independently completed forward translation from English to Modern Standard Arabic (MSA). The two versions were synthesized into one version, which was then back translated from Arabic to English by two different students. These back translated versions were compared to the original version to check for discrepancies and refine the Arabic translation. The translated version was then reviewed independently by three local experts with knowledge of the target community and the constructs measured in the questionnaires (e.g., clinical psychologists working with Syrian refugees). Where necessary, the MSA version was supplemented with alternative dialect words to improve comprehensibility.

Following translation, the questionnaires were piloted during focus group discussions with Syrian children and caregivers, and then during a series of pilot studies (sample size N = 30-100 for each questionnaire). This was used to further refine questions (by adjusting language or providing examples) and to guide modifications to the scales (deciding which items to remove when abridging scales). The refined versions were then further pilot tested to ensure modified questions were clear. All interviewers were trained on all aspects of data collection, including communication with children, research ethics, the specific measurement tools, and how to adjust phrasing of questions for different Arabic dialects. See Table S1 for details of how specific measures were modified.

The Strengths and Difficulties Questionnaire (SDQ) was not modified due to licencing restrictions: the published Arabic version was used in this case.

1.2 Mental Health Outcome Cut-Off Scores

Optimal cut-off scores for mental health outcome measures were derived from a subsample (n=119) of the BIOPATH cohort who completed clinical interviews. Consensus clinical diagnosis was based on the MINI-Kid structured clinical interview, a Clinical Global Impression severity (CGI-s) score, and clinical judgement following supervision with an experienced clinical psychologist. Questionnaires (CES-DC, CPSS, SCARED, SDQ, CD/ODD items) were administered on the same day (n = 101) or on a different day (n = 18; median gap = 20 days). Receiver operating characteristic curve (ROC curve) analysis was carried out to identify the cut-off that achieved the best balance between sensitivity and specificity. Results are reported in McEwen et al. (2020). To measure externalising behaviour problems, we used a composite of the SDQ externalising scale and the additional

CD/ODD items (total score 0-44). The optimal cut off on the composite was then determined by ROC curve analysis as above (AUC = .81, $p < .001$; sensitivity = .85, specificity = .71, correctly classified = 75.4%).

1.3 Table S1. Details of Measures

Measure	Child or caregiver report	Instrument	Description/modifications	Rationale for inclusion
Outcomes				
Post-traumatic stress	Child	Child PTSD Symptom Scale (CPSS) (Foa *et al.* 2001)	Based on DSM-IV criteria for PTSD; instructions supplemented to clarify the types of events probed for and timing of events; clarification added to one symptom item; functional impairment scale not used; items added to probe for past episodes (not used in total symptom score)	Outcome Measure
Depression	Child	Centre for Epidemiological Studies Depression Scale for Children (CES-DC) (Abdo 2016; Faulstich *et al.* 1986; Weissman *et al.* 1980)	Reduced to 10 items following piloting; items added to probe for past episodes (not used in total symptom score)	Outcome Measure
Externalising behaviour problems	Caregiver	Strengths and Difficulties Questionnaire (SDQ): Externalising score (Alyahri & Goodman, 2006; Goodman, Lamping, & Ploubidis, 2010; Goodman, 1997)	SDQ administered in its entirety as published; conduct and hyperactivity subscales used as measure of externalising behaviour problems	Outcome Measure
		Items aligned with DSM-5 conduct disorder and oppositional defiant disorder criteria (McEwen *et al.* 2022)	12 items developed for this study, omitting sensitive areas (forced sexual activity) and more severe behaviours (e.g., firesetting, use of a gun)	Outcome Measure
Wellbeing	Child	World Health Organisation – Five Wellbeing Index (WHO-5; Bech 2012; Sibai *et al.* 2009; Topp *et al.* 2015)	Minor changes to translation to improve comprehensibility	Outcome Measure

Measure	Child or caregiver report	Instrument	Description/modifications	Rationale for inclusion
Covariates				
Age	Child		Age at baseline, rounded to the nearest year	Older children and adolescents tend to display more PTSD and depression symptoms and less externalising, although some find age protective against internalising, and others find no relationship (Scharpf *et al.* 2021); some evidence that age can interact with other factors to influence e.g. peer relationships (Zevulun *et al.* 2017)
Gender	Child		Binary male/female variable	Girls tend to be at more risk of internalising and PTSD symptoms, boys more at risk of externalising symptoms (Scharpf *et al.* 2021)
Time since leaving Syria	Caregiver		Five categories: 0-12 months, 12-24 months, 24-36 months, 36-48 months, > 48 months ago	Longer time since displacement sometimes related with better mental health, but in some circumstances (e.g. camps) symptoms worsen (Scharpf *et al.* 2021)
War exposure	Child & caregiver	War Events Questionnaire (Karam *et al.* 1999)	Combined checklist of events completed by child and caregiver about child's war exposure	Cumulative war exposure associated with higher PTSD, depression, & externalising in most studies of refugee youth (Scharpf *et al.* 2021)
Predictors				

Measure	Child or caregiver report	Instrument	Description/modifications	Rationale for inclusion
Optimism	Child	Youth Life Orientation Test (YLOT) (Ey *et al.* 2005)	Four optimism items were selected, dropping two that might be less indicative of optimism in a refugee setting	Associated with fewer internalising and externalising (Speidel *et al.* 2021) and PTSD (Sleijpen *et al.* 2016) symptoms in refugees in high income countries, but lack evidence in high risk context
Self-efficacy	Child	General Self-Efficacy Scale (GSE) (Schwarzer & Jerusalem 1995)	Selected four items most suitable for children; replaced one following piloting	Theoretically key element determining ability to adapt to change (Brown & Westaway 2011), evidence both for (Chung *et al.* 2017) and against (Van Heemstra *et al.* 2021) protective effect in adult refugees, but lack evidence in child refugees
Self-esteem	Child	Lifespan Self-Esteem Scale (LSE) (Harris *et al.* 2018)	Selected one item following piloting (all 4 original items were perceived to mean the same)	Although self-esteem can be treated as an outcome (Scharpf *et al.* 2021), there is evidence to suggest it can reflect a stable trait (Brent Donnellan *et al.* 2012); some evidence it can act as a protective factor in refugee children (Marley & Mauki 2019)
Child religiosity	Child	Items from NICHD Study of Early Child Care and Youth Development, and National Study of Youth and Religion Survey (NSYRS) (Barber 2001; Kessler & Ustün 2004; Pearce *et al.* 2013)	Removed an item on attending religious meetings to avoid gender bias	Evidence as a protective factor in refugee children (Arakelyan & Ager 2021), but in qualitative research adolescents place varying degrees of importance (Nagi *et al.* 2021), and results vary by outcome (Tol *et al.* 2013)
Future orientation	Child			

Measure	Child or caregiver report	Instrument	Description/modifications	Rationale for inclusion
Future aspirations<br>Future expectations<br>Future planning and motivation		Future Aspirations and Plans (FAP) (McEwen *et al.* 2022)	New measure developed for this study, based on i) focus groups with Syrian refugee children in Lebanon; ii) Lindstrom Johnson et al. (2014) future orientation framework (Johnson *et al.* 2014); iii) items adapted from Consideration of Future Consequences scale (Strathman *et al.* 1994)	Mediates between traumatic events and mental health in children (Zhang *et al.* 2009), and qualitative reports from refugees emphasise future orientation (Nyarko & Punamaki 2020), but quantitative evidence is lacking in refugee populations
Environmental sensitivity	Child	Highly Sensitive Child Scale (HSC) (Pluess *et al.* 2018)	12 item version used; modified three items following pilot testing to reduce possible social desirability effects	Theoretically should determine strength of environmental influence (Pluess 2015); interacts with adversity to predict effects of war on PTSD (Karam et al., 2019), but only preliminary research without evidence on other mental health outcomes in refugees
Coping strategies<br>Problem-focused coping<br>Cognitive restructuring coping<br>Distraction coping<br>Avoidance coping<br>Support-seeking coping	Child	Children's Coping Strategies Checklist (CCSC) (Program for Prevention Research 1999)	Reduced to 15 items, 3 per subscale, based on pilot testing	Problem-focused coping, cognitive restructuring, and support-seeking generally protective while distraction and avoidance generally negative (Arakelyan & Ager 2021; Scharpf *et al.* 2021), but in certain situations problem-focused coping associated with worse PTSD (Elklit *et al.* 2012), and the association between avoidance and psychological symptoms might be explained by PTSD avoidance symptoms (McGregor *et al.* 2015)

Measure	Child or caregiver report	Instrument	Description/modifications	Rationale for inclusion
Maternal acceptance	Child	Acceptance subscale - Child Report of Parent Behavior Inventory (CRPBI) (Schaefer 1965)		Positive family environment consistently reported protective factor (Arakelyan & Ager 2021), adolescent refugees highlight maternal support in particular (Nagi *et al.* 2021)
Positive home experiences	Child	Positive Home Experiences (PHE) (McEwen *et al.* 2022)	Four items asking about presence of parents during day to day activities	Quality time spent with parents associated with child wellbeing (Suldo & Fefer 2013) and child refugees separated from family members at higher risk (Scharpf *et al.* 2021), but lack evidence on day to day parental presence in refugees
Parental monitoring	Child	Parental behavioural control (Barber 1996)		Associated with fewer problems and more positive outcomes in war-exposed children (Eltanamly *et al.* 2021)
Perceived social support	Child	Multidimensional Scale of Perceived Social Support for Arab American Adolescents (MSPSS-AA) (Ramaswamy *et al.* 2009)	Subscales for social support from family and friends; used 5-point rating scale instead of 3-point scale	Support from family, friends, and general support is protective, but findings differ across studies (Scharpf *et al.* 2021)
Maternal psychological control	Child	Psychological Control – Disrespect Scale (PCDS) (Barber *et al.* 2012)		Consistently associated with worse internalising and externalising problems in refugee children (Scharpf *et al.* 2021)
Parent-child conflict	Child	Parent-adolescent conflict (Barber 1999)	Response options were reduced from 6 to 5 and reworded to be consistent with items on positive home experiences	Conflicts with family members can be associated with worse outcomes in refugees, particularly when the disagreement is related to acculturation (Scharpf *et al.* 2021)

Measure	Child or caregiver report	Instrument	Description/modifications	Rationale for inclusion
Child maltreatment	Child	ISPCAN Child Abuse Screening Tool (ICAST) (Runyan *et al.* 2009)	Shortened to 22 items; sexual abuse items removed due to sensitivity and replaced by item enquiring about "private events" (the child was not required to disclose the details)	Associated with PTSD, depression, and some externalising symptoms in refugee populations (Scharpf *et al.* 2021)
Bullying	Child	Bullying of Refugee Children (BRC) (McEwen *et al.* 2022)	8 item scale developed for this study based on advice from agencies working with Syrian refugee children in Lebanon about types of victimisation commonly experienced and modified following piloting	Associated with worse outcomes, but evidence is from children in school (Çeri *et al.* 2021; Samara *et al.* 2020), whilst the majority of our sample are not in school
Loneliness and social isolation	Child	Loneliness in Refugee Children (LRC) (Asher *et al.* 1984; McEwen *et al.* 2022)	Two items adapted from Loneliness and Social Dissatisfaction Scale Items; two items were written to capture social isolation related to refugee context	Modifies effects of pre-displacement adversity on mental health (Chen *et al.* 2017) but lack evidence in refugee children
Caregiver depression	Caregiver	Center for Epidemiologic Studies Short Depression Scale (CES-D 10) (Radloff 1977)	Items added to probe for past episodes (not used in total symptom score)	Parent mental health consistently associated with refugee child mental health (Scharpf *et al.* 2021), most evidence for maternal caregivers (Javanbakht *et al.* 2018), but some inconsistent findings of effects on different dimensions of children's mental health (Eruyar *et al.* 2018)
Caregiver PTSD	Caregiver	The PTSD Checklist for DSM-5 (PCL-5) (Blevins *et al.* 2015)	Items added to probe for past episodes (not used in total symptom score)	As above
Caregiver anxiety	Caregiver	Depression Anxiety and Stress Scale (DASS-21) Anxiety subscale (Henry & Crawford 2005)	Items added to probe for past episodes (not used in total symptom score)	As above

Measure	Child or caregiver report	Instrument	Description/modifications	Rationale for inclusion
Human insecurity	Caregiver	Human Insecurity Scale (Ziadni *et al.* 2011)		Post-migration stressors experienced by caregivers associated with child mental health (Scharpf *et al.* 2021) and some evidence insecurity is associated with internalising in displaced youth (Betancourt *et al.* 2012)
Perceived refugee environment	Caregiver	Perceived Refugee Environment Index (PREI) (McEwen *et al.* 2022)	Developed for this study as multidimensional measure to assess the quality of the refugee environment; subscales: livelihood, basic needs, housing, access to services, family environment, community environment, working situation, future mobility [not included in total score], learning environment	Aspects of the physical and social environment often relate to mental health, some findings are inconsistent (Scharpf *et al.* 2021), but the environment may be particularly important in camp settings (Arakelyan & Ager 2021)
Collective efficacy	Caregiver	Collective Efficacy (Sampson *et al.* 1997)		Social aspects of the community play distinct roles in child mental health compared to other social support (Arakelyan & Ager 2021)
Access to education	Child & Caregiver	Single item 'No school/Some education/Attends school' (McEwen *et al.* 2022)	Created from child single item 'Do you go to school?' combined with caregiver PREI school items	No (Scherer *et al.* 2020) or interrupted (Nasıroğlu *et al.* 2018) education is a risk factor for refugee children; education is central to wellbeing (Arakelyan & Ager 2021)
Parent abroad	Child	Single item 'Does either of your parents live most of the time in another country?'		Integrity of family unit important, but some studies identify the presence of at least one biological parent is protective (Arakelyan & Ager 2021; Scharpf *et al.* 2021)

Measure	Child or caregiver report	Instrument	Description/modifications	Rationale for inclusion
Child responsibilities	Child	Sum of the time child spent doing house chores, caring for family and livestock, work in the fields, other work outside the home, and seasonal jobs		Work in this context is often dangerous (Habib *et al.* 2019); child labour in other refugee populations associated with higher odds of depression, but opposite pattern for anxiety (Meyer *et al.* 2020)
Parent deceased	Caregiver	Single item 'Spouse/child's other parent deceased'		Integrity of family unit important, but some studies identify the presence of at least one biological parent is protective (Arakelyan & Ager 2021; Scharpf *et al.* 2021)
Household size	Caregiver	Sum of total number of adults and children in the household.		Some studies find no effect (Scharpf *et al.* 2021), but others show evidence for protective effect of larger family (Scherer *et al.* 2020; Wiegersma *et al.* 2011)
Family income	Caregiver	Single item 'Family income per week'		Current economic status and poverty important for outcomes (Arakelyan & Ager 2021; Scharpf *et al.* 2021)
Caregiver currently working	Caregiver	Single item 'Do you have a job?'		Paternal unemployment linked to child psychopathology (Sapmaz *et al.* 2017) but lack evidence on mothers
Household adult literacy	Caregiver	Single item 'Can parent/household adults read and write?'		Some evidence for association of parental literacy and level of education with child symptoms in certain populations but not others (Arakelyan & Ager 2021; Scharpf *et al.* 2021)

Measure	Child or caregiver report	Instrument	Description/modifications	Rationale for inclusion
Child general health	Caregiver	Single item ‘Excellent – Very poor’	Higher scores indicate worse general health	Worse physical health associated with worse mental health in refugee children (Lau *et al.* 2018)
Caregiver general health	Caregiver	Single item ‘Excellent – Very poor’	Higher scores indicate worse general health	Mental and physical health strongly linked (Berthold *et al.* 2014) and caregiver wellbeing associated with child outcomes (Scharpf *et al.* 2021)

Note. Full descriptions of measures can be found in the cohort profile (McEwen *et al.* 2022)

1.4 Figure S1. Cross-Lagged Panel Models: Full Model Illustration

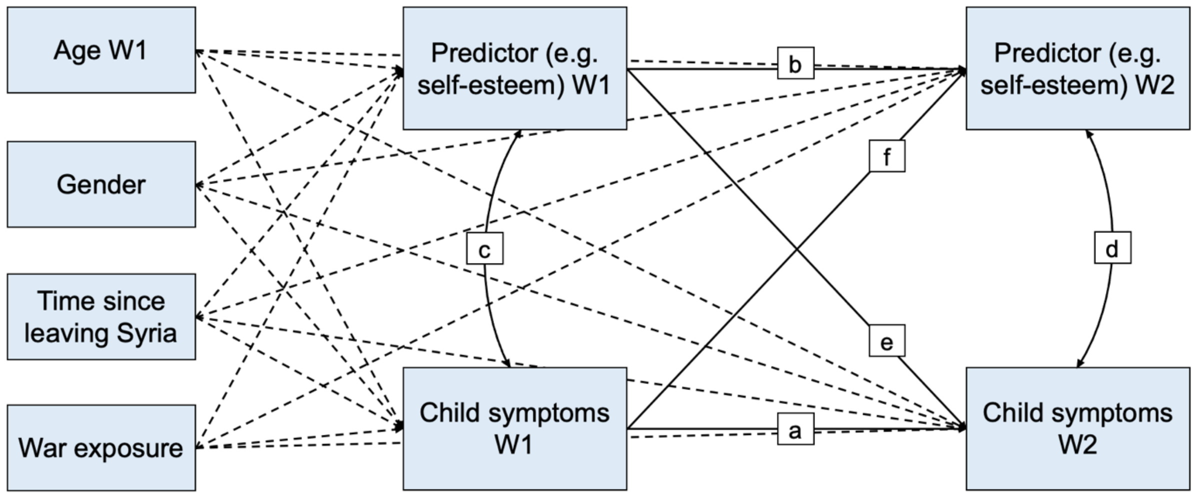

Note. Structural illustration of the full cross-lagged panel models tested, including the pathways controlling for age, gender, time since leaving Syria, and war exposure. Pathways: a = child symptom auto-regressed pathway; b = predictor auto-regressed pathway; c = W1 covariance; d = W2 covariance; e = cross lagged pathway: predictor → symptoms; f = cross lagged pathway: symptoms → predictor.

W1 = baseline, W2 = follow-up

2. Supplemental Results

2.1 Comparison of Current Sample to Baseline

Our sample did not differ from the original baseline sample in terms of child or caregiver gender, caregiver age, nationality, child marriage, or household size. In families included in our analyses, children were slightly younger than those not included ($t(1201.9) = 4.68$, $p < .001$, $d = 0.25$), more likely to participate with their mother ($\chi^2(11) = 29.48$, $p = .002$, *tau* = .004), to have left Syria more than three years before recruitment ($\chi^2(1) = 20.65$, $p < .001$, $d = .12$), to be registered with UNHCR ($\chi^2(2) = 19.92$, $p < .001$, $d = -0.07$), and to have access to education ($\chi^2(2) = 24.77$, $p < .001$, $d = 0.14$). They were less likely to be from the most vulnerable localities ($\chi^2(2) = 28.97$, $p < .001$, $d = 0.10$). However, all these differences were of small effect size.

2.2 Table S2. Analyses of Covariance: Main Results from all Models

Factor		Mental health risk group M(SD)				F	Adj. R Square	Post-hoc comparisons
		Stable High Risk (SHR)	Deteriorating (D)	Improving (I)	Stable Low Risk (SLR)			
Optimism	Baseline	8.82 (3.14)	9.65 (2.51)	9.5 (2.73)	9.87 (2.43)	9.71***	0.42	SHR < D / I / SLR
	Change	0.45 (4.21)	0.2 (3.93)	0.71 (3.66)	0.67 (3.27)	6.03**	0.40	SHR < I / SLR
Self-efficacy	Baseline	11.12 (3.46)	11.5 (3.45)	11.5 (3.58)	11.33 (3.44)	0.65		
	Change	0.91 (4.64)	0.52 (4.95)	0.51 (4.46)	1.3 (4.06)	1.20		
Self-esteem	Baseline	3.88 (1.27)	4.41 (0.64)	4.13 (1.04)	4.44 (0.69)	10.76***	0.47	SHR < D / I / SLR
	Change	0.1 (1.41)	-0.3 (1.04)	0.21 (1.15)	-0.07 (0.99)	5.86**	0.41	SHR / D < I / SLR
Environmental sensitivity	Baseline	5.1 (1.01)	4.76 (0.98)	5.07 (1.05)	4.87 (0.93)	6.78***	0.52	I / SLR < SHR
	Change	-0.23 (1.34)	0.16 (1.31)	-0.56 (1.24)	-0.26 (1.21)	10.97***	0.50	I / SLR < SHR / D
Religiosity	Baseline	31.45 (6.37)	32.17 (6.6)	32.25 (6.7)	31.29 (5.98)	1.49		
	Change	2.28 (8.15)	2.11 (8.12)	1.86 (7.91)	2.81 (7.29)	0.17		
Future aspirations	Baseline	3.28 (1.08)	3.36 (0.99)	3.23 (1.09)	3.23 (1.14)	1.60		
	Change	0.09 (1.35)	0.02 (1.26)	-0.08 (1.44)	0.01 (1.38)	2.07		
Future expectations	Baseline	2.98 (1.04)	2.98 (0.98)	3.07 (0.97)	2.94 (0.99)	1.01		
	Change	0.16 (1.33)	0.01 (1.17)	-0.12 (1.32)	0.1 (1.15)	2.87		
Future planning	Baseline	3.03 (0.9)	3.05 (0.78)	3.01 (0.88)	2.94 (0.95)	2.01		

Factor		Mental health risk group M(SD)				F	Adj. R Square	Post-hoc comparisons
		Stable High Risk (SHR)	Deteriorating (D)	Improving (I)	Stable Low Risk (SLR)			
	Change	0.05 (1.19)	-0.08 (1.11)	-0.22 (1.22)	0.03 (1.19)	4.95**	0.40	I < SHR
Problem-focused coping	Baseline	7.91 (2.75)	7.31 (2.6)	8.08 (2.72)	6.97 (2.58)	1.73		
	Change	0.83 (3.71)	1.52 (3.79)	0.19 (3.69)	1.04 (3.46)	2.26		
Avoidant coping	Baseline	7.91 (2.75)	7.31 (2.6)	8.08 (2.72)	6.97 (2.58)	1.48		
	Change	0.27 (3.72)	1 (4.01)	-0.16 (3.86)	1.08 (3.42)	1.00		
Support seeking	Baseline	6.48 (3.06)	5.73 (2.71)	6.25 (2.94)	5.78 (2.89)	1.10		
	Change	0.42 (4.35)	1.13 (3.97)	0.56 (3.89)	1.17 (4.14)	0.06		
Distraction coping	Baseline	6.45 (2.3)	6.45 (2.19)	6.45 (2.29)	6.38 (1.88)	2.78		
	Change	-0.71 (3.16)	-1.16 (2.71)	-0.26 (3.11)	-0.06 (2.59)	6.20**	0.42	SHR / D < I / SLR
Positive cognitive restructuring	Baseline	8.18 (2.7)	8.36 (2.66)	8.51 (2.68)	7.9 (2.65)	2.92		
	Change	0.16 (3.93)	-0.23 (3.66)	0.39 (3.48)	0.59 (3.67)	2.32		
Child general health	Baseline	2.37 (0.82)	2.13 (0.91)	2.28 (0.79)	2.12 (0.73)	7.00***	0.47	I / SLR < SHR
	Change	-0.31 (1.01)	-0.08 (1.19)	-0.48 (0.94)	-0.35 (0.92)	7.45***	0.46	I / SLR < SHR / D
Bullying	Baseline	5.7 (6.83)	3.18 (5.31)	3.98 (5.79)	2.78 (4.54)	12.52***	0.50	D / I / SLR < SHR
	Change	-1.23 (7.93)	0.58 (6.68)	-1.94 (7.09)	-0.82 (5.55)	8.03***	0.45	I < SHR / D<br>SLR < SHR

Factor		Mental health risk group M(SD)				F	Adj. R Square	Post-hoc comparisons
		Stable High Risk (SHR)	Deteriorating (D)	Improving (I)	Stable Low Risk (SLR)			
Loneliness and social isolation	Baseline	8.72 (3.02)	7.02 (2.37)	8.23 (2.69)	7.01 (2.49)	23.77***	0.50	D < SHR<br>SLR < I < SHR
	Change	-0.96 (4.12)	-0.19 (4.05)	-1.61 (3.78)	-0.88 (3.39)	12.65***	0.47	I < SHR<br>SLR < D < SHR
Perceived social support	Baseline	5.5 (0.97)	5.68 (0.8)	5.61 (0.81)	5.72 (0.76)	3.27*	0.37	SHR < D / I / SLR
	Change	0.13 (1.31)	0.22 (1.2)	0.19 (1.05)	0.13 (1.03)	2.40		
Maternal acceptance	Baseline	26.79 (4.4)	27.82 (3.18)	27.41 (3.66)	28.28 (2.61)	7.90***	0.34	SHR < D / I / SLR
	Change	0.24 (5.39)	-0.04 (4.09)	0.63 (4.54)	0.52 (3.54)	3.69*	0.32	SHR < I / SLR
Maternal psychological control	Baseline	11.61 (2.86)	10.52 (1.5)	10.86 (2.05)	10.3 (1.65)	14.96***	0.49	D / I / SLR < SHR
	Change	-0.66 (3.29)	-0.31 (1.98)	-0.6 (2.21)	-0.13 (2.23)	4.68**	0.47	SHR < D / I / SLR
Parent-child conflict	Baseline	6.12 (3.25)	5.74 (2.97)	5.93 (3.29)	5.06 (2.33)	8.23***	0.37	SLR / I < SHR<br>SLR < D
	Change	1.72 (4.97)	1.34 (4.53)	0.53 (4.52)	1.09 (3.8)	9.10***	0.38	I / SLR < SHR
Parental monitoring	Baseline	13.88 (2.14)	14.23 (1.26)	14.1 (1.95)	14.21 (2.04)	3.02		
	Change	-0.01 (2.87)	0.05 (1.75)	0.27 (2.18)	0.01 (2.06)	2.52		
Positive home	Baseline	15.88 (3.83)	15.11 (3.89)	16.04 (3.93)	15.53 (3.72)	0.21		

Factor		Mental health risk group M(SD)				F	Adj. R Square	Post-hoc comparisons
		Stable High Risk (SHR)	Deteriorating (D)	Improving (I)	Stable Low Risk (SLR)			
experiences	Change	-0.5 (5.36)	1.23 (5.5)	-0.6 (5.3)	0.56 (4.78)	1.78		
Child maltreatment	Baseline	13.82 (13.52)	7.46 (8.27)	11.08 (11.81)	7.23 (8.21)	20.30***	0.50	I < SHR<br>SLR < D < SHR
	Change	-3.09 (15.1)	1.41 (10.84)	-5.99 (13.63)	-3.3 (10.11)	17.13***	0.48	I / SLR < SHR / D
Caregiver depression	Baseline	16.19 (6.35)	12.44 (6.33)	15.2 (6.33)	13.12 (6.43)	24.10***	0.36	SLR < I / D < SHR
	Change	-0.31 (7.87)	1.46 (7.71)	-4.52 (8.64)	-3.79 (8.26)	37.93***	0.36	I / SLR < SHR / D
Caregiver PTSD	Baseline	35.95 (17.33)	27.64 (14.62)	34.93 (17.9)	29.01 (18.25)	14.12***	0.47	SLR < D < SHR<br>I < SHR
	Change	-8.08 (22.39)	-2.42 (20.7)	-17.75 (25.19)	-13.04 (21.22)	23.82***	0.45	I / SLR < SHR / D
Caregiver anxiety	Baseline	8.69 (5.39)	6.25 (4.83)	8.36 (5.34)	7.28 (5.36)	9.35***	0.43	D / I / SLR < SHR
	Change	-0.88 (6.58)	0.13 (5.72)	-3.08 (6.56)	-2.34 (6.89)	14.30***	0.41	I / SLR < SHR / D
Caregiver general health	Baseline	3.04 (0.9)	2.84 (0.97)	2.97 (0.92)	2.8 (0.93)	4.12*	0.37	I / SLR < SHR
	Change	-0.18 (1.06)	-0.07 (1.07)	-0.41 (1.06)	-0.09 (1.13)	6.00**	0.37	I < SHR / D<br>SLR < I
Human insecurity	Baseline	3.7 (0.37)	3.64 (0.41)	3.67 (0.43)	3.7 (0.42)	5.08**	0.51	I < SHR
	Change	0.08 (0.47)	0.15 (0.51)	-0.02 (0.61)	-0.03 (0.6)	7.71***	0.51	I / SLR < SHR / D

Factor		Mental health risk group M(SD)				F	Adj. R Square	Post-hoc comparisons
		Stable High Risk (SHR)	Deteriorating (D)	Improving (I)	Stable Low Risk (SLR)			
Perceived refugee environment	Baseline	3.21 (0.51)	3.27 (0.53)	3.28 (0.5)	3.14 (0.51)	9.39***	0.46	SHR / D / SLR < I
	Change	0.03 (0.6)	0.04 (0.61)	0.21 (0.61)	0.29 (0.63)	15.64***	0.42	SHR / D < I / SLR
Collective efficacy	Baseline	31.9 (6.09)	33.04 (6.25)	32.63 (6.49)	32.72 (6.72)	2.07		
	Change	-0.78 (9.19)	-0.8 (10.22)	-0.13 (9.44)	-1.12 (10.58)	1.29		
Household size	Baseline	7.56 (2.51)	7.5 (2.33)	7.82 (2.42)	7.81 (2.46)	1.04		
	Change	-0.02 (2.06)	0.16 (1.97)	0.14 (2.22)	-0.02 (1.95)	0.81		
School attendance	Baseline	1.1 (0.85)	1.04 (0.89)	1.02 (0.88)	0.86 (0.86)	3.09		
	Change	-0.35 (0.85)	-0.29 (0.91)	-0.4 (0.84)	-0.2 (0.91)	1.34		
Parent abroad	Baseline	0.15 (0.36)	0.06 (0.24)	0.15 (0.36)	0.09 (0.29)	1.35		
	Change	-0.03 (0.37)	0.02 (0.32)	-0.01 (0.39)	0.01 (0.28)	0.14		
Child responsibilities	Baseline	4.41 (3.49)	3.62 (2.81)	4.22 (3.28)	4.18 (2.75)	1.97		
	Change	1.08 (4.09)	1.76 (3.91)	0.47 (4.11)	1.2 (4.06)	4.48**	0.36	I < SHR / D
Parent deceased	Baseline	0.08 (0.27)	0.09 (0.29)	0.08 (0.27)	0.04 (0.21)	0.29		
	Change	0.01 (0.21)	-0.02 (0.24)	0 (0.15)	0.01 (0.11)	0.38		
Caregiver employed	Baseline	1.88 (0.32)	1.89 (0.31)	1.88 (0.33)	1.92 (0.27)	0.90		
	Change	-0.08 (0.45)	-0.07 (0.41)	-0.02 (0.44)	-0.06 (0.46)	1.37		

Factor		Mental health risk group M(SD)				F	Adj. R Square	Post-hoc comparisons
		Stable High Risk (SHR)	Deteriorating (D)	Improving (I)	Stable Low Risk (SLR)			
Family income	Baseline	1.86 (1.08)	1.95 (1.05)	1.93 (1.11)	1.79 (1.04)	0.18		
	Change	0.12 (1.53)	0.06 (1.25)	0.05 (1.57)	0.25 (1.26)	0.14		
Adult literacy	Baseline	2.6 (1.17)	2.59 (1.15)	2.53 (1.16)	2.48 (1.17)	0.93		
	Change	0.14 (1.49)	-0.18 (1.35)	0.05 (1.3)	0.07 (1.55)	2.24		

Note. Table representing descriptive statistics and analyses of covariance (ANCOVAs) from all predictors using imputed data (N = 982). Child age, gender, time since leaving Syria, and war exposure were entered as covariates into all ANCOVAs. Baseline models controlled for change scores, and change models controlled for baseline scores. F statistic is based on test against null model including only covariates. Adjusted R Square is based on full model. Post-hoc comparisons are based on Tukey's test. Means and SDs are unadjusted estimates, all other statistics are based on adjusted means according to the ANCOVA models. Higher scores on child and caregiver general health indicate worse health. P-values based on Benjamini-Hochberg correction for multiple testing (Benjamini & Hochberg 1995). * $p < .05$, ** $p < .01$, *** $p < .001$

2.3 Figure S2. Cross-Lagged Panel Models: Main Results

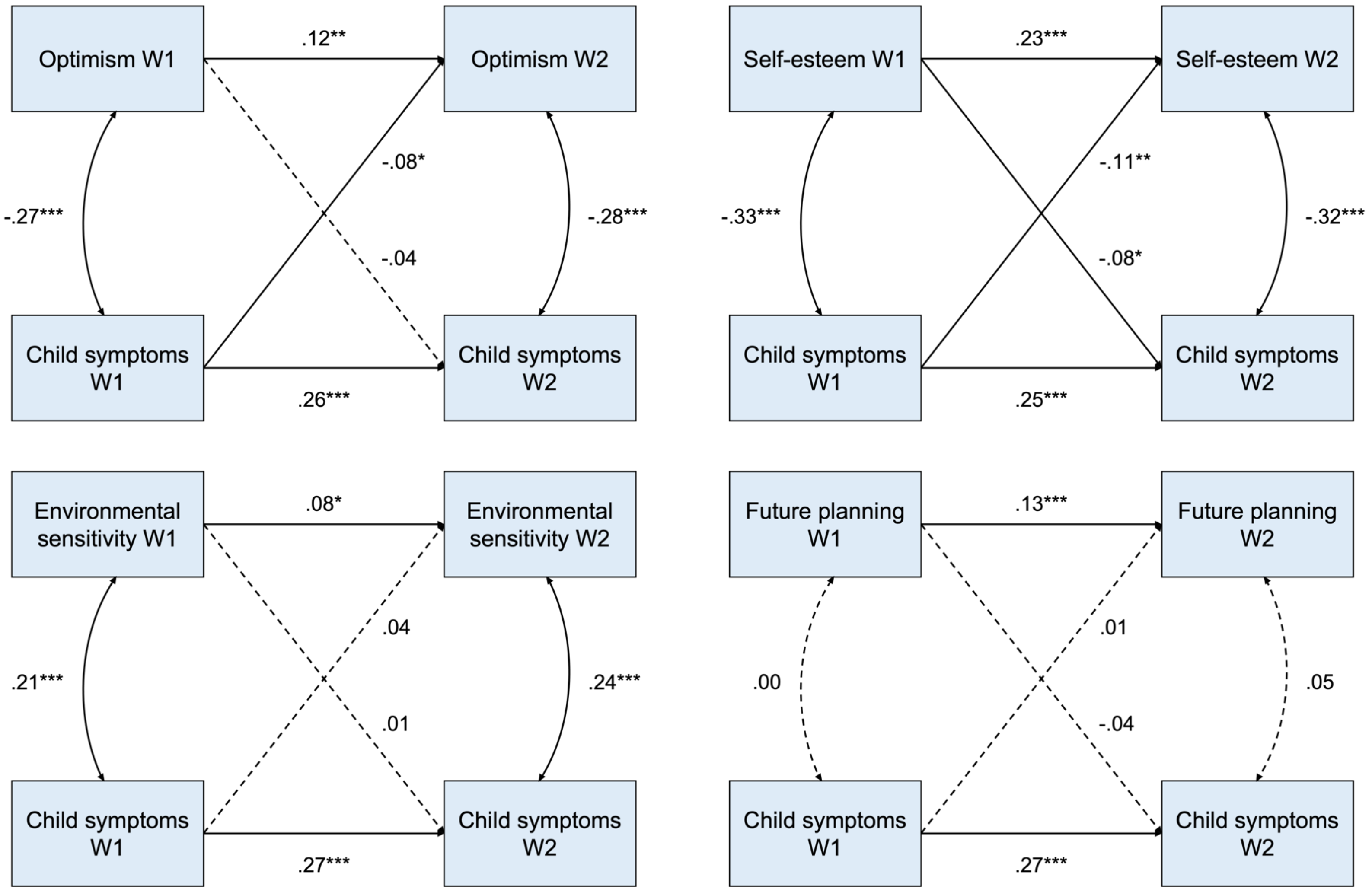

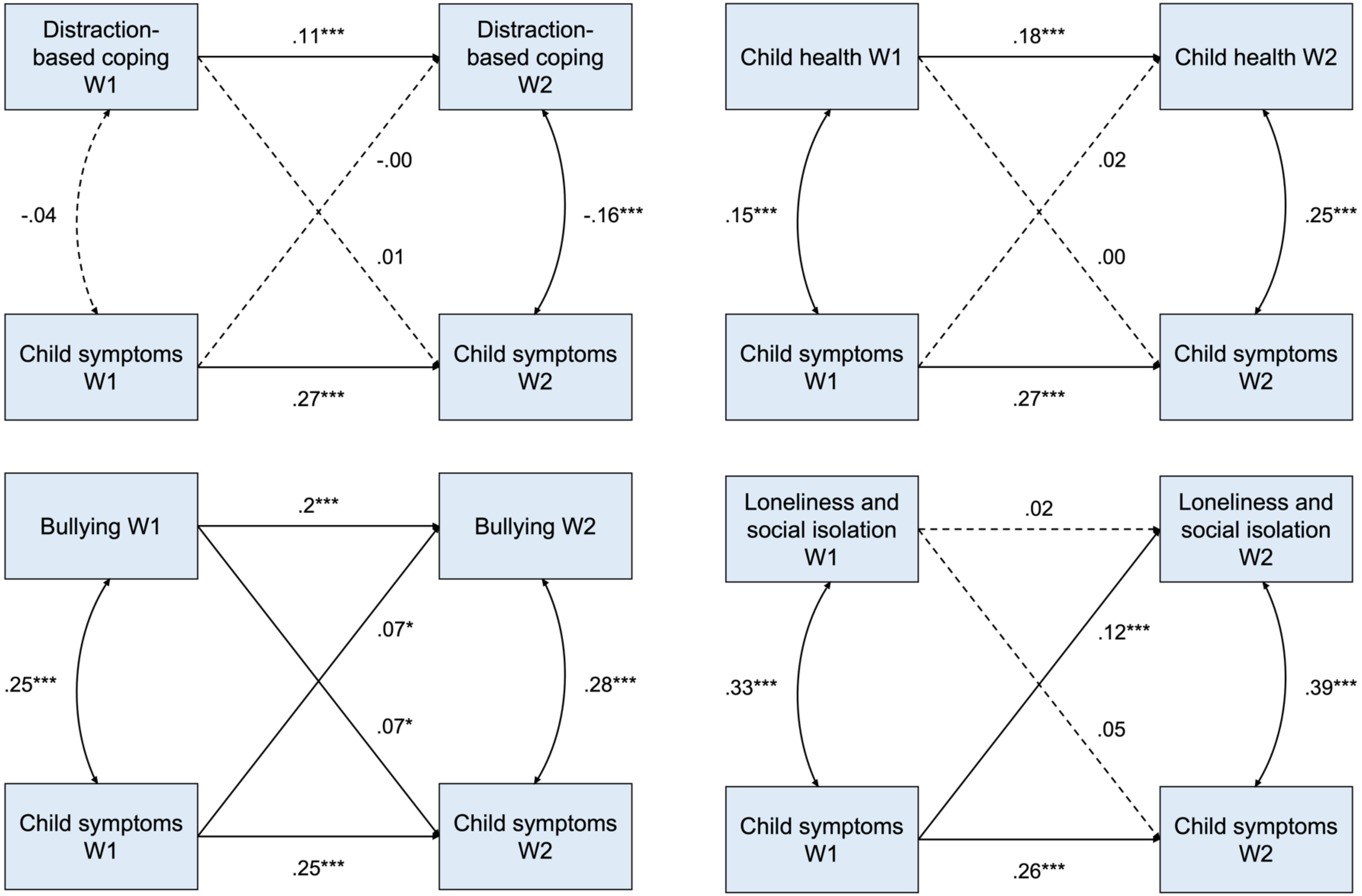
Distraction-based coping W1
Distraction-based coping W2
.11***
-.04
-.00
.01
-.16***
Child symptoms W1
Child symptoms W2
.27***
Child health W1
Child health W2
.18***
.15***
.02
.00
.25***
Child symptoms W1
Child symptoms W2
.27***
Bullying W1
Bullying W2
.2***
.25***
.07*
.07*
.28***
Child symptoms W1
Child symptoms W2
.25***
Loneliness and social isolation W1
Loneliness and social isolation W2
.02
.33***
.12***
.05
.39***
Child symptoms W1
Child symptoms W2
.26***

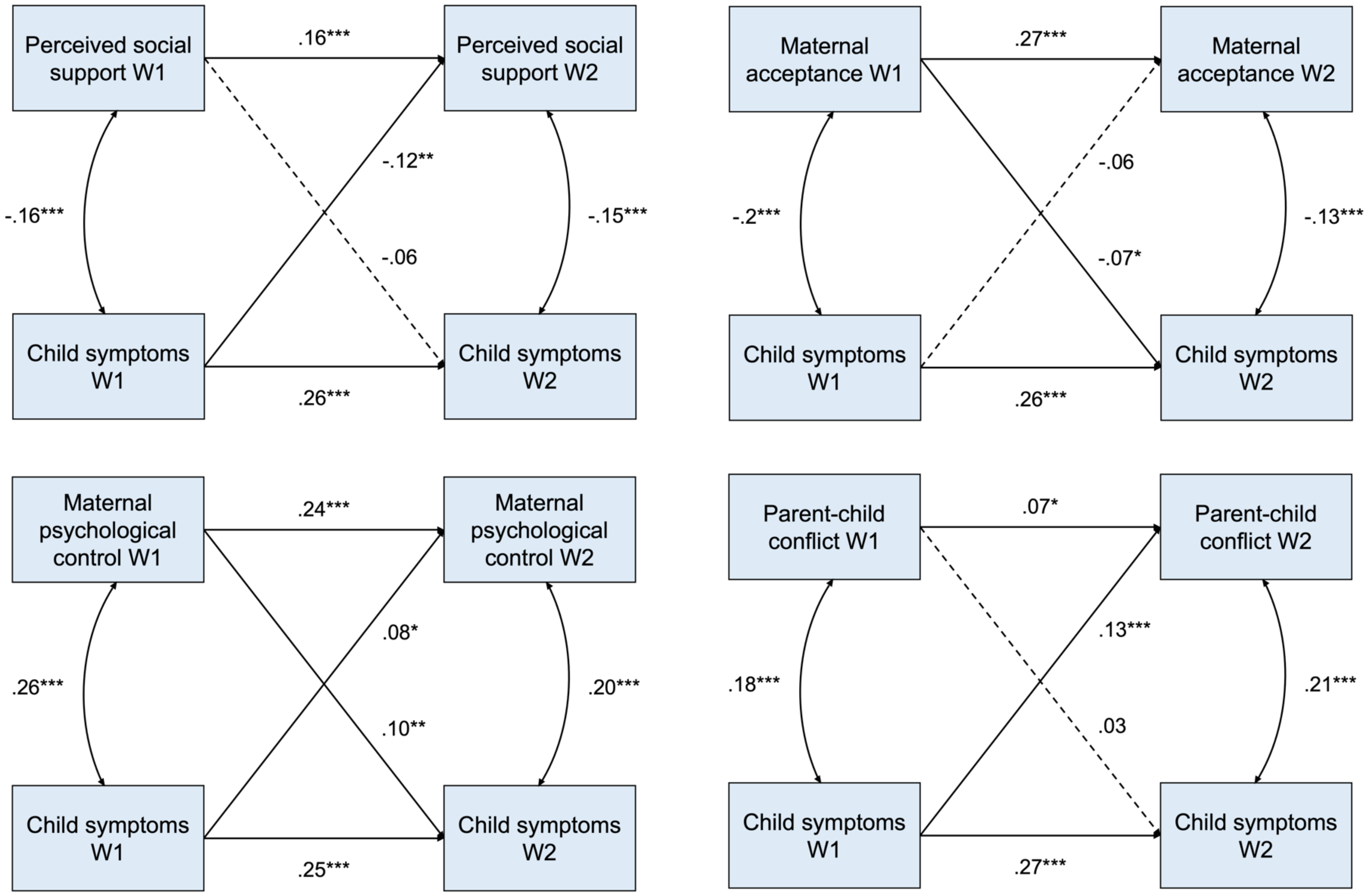
Perceived social support W1
Perceived social support W2
Child symptoms W1
Child symptoms W2
.16***
-.16***
-.12**
-.06
-.15***
.26***
Maternal acceptance W1
Maternal acceptance W2
Child symptoms W1
Child symptoms W2
.27***
-.2***
-.06
-.07*
-.13***
.26***
Maternal psychological control W1
Maternal psychological control W2
Child symptoms W1
Child symptoms W2
.24***
.26***
.08*
.10**
.20***
.25***
Parent-child conflict W1
Parent-child conflict W2
Child symptoms W1
Child symptoms W2
.07*
.18***
.13***
.03
.21***
.27***

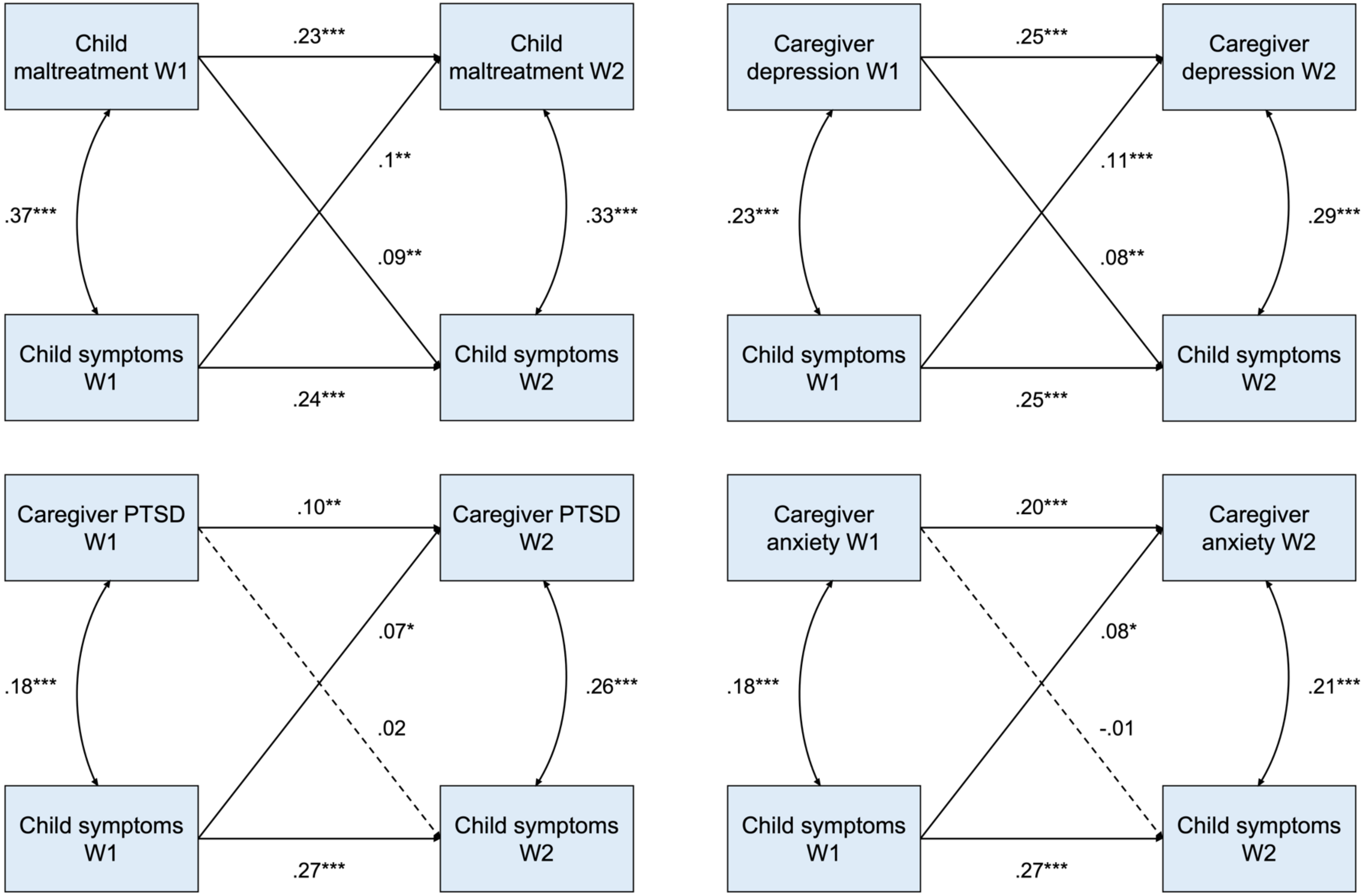
Child maltreatment W1
.23***
Child maltreatment W2
.37***
.1**
.09**
.33***
Child symptoms W1
.24***
Child symptoms W2
Caregiver depression W1
.25***
Caregiver depression W2
.23***
.11***
.08**
.29***
Child symptoms W1
.25***
Child symptoms W2
Caregiver PTSD W1
.10**
Caregiver PTSD W2
.18***
.07*
.02
.26***
Child symptoms W1
.27***
Child symptoms W2
Caregiver anxiety W1
.20***
Caregiver anxiety W2
.18***
.08*
-.01
.21***
Child symptoms W1
.27***
Child symptoms W2

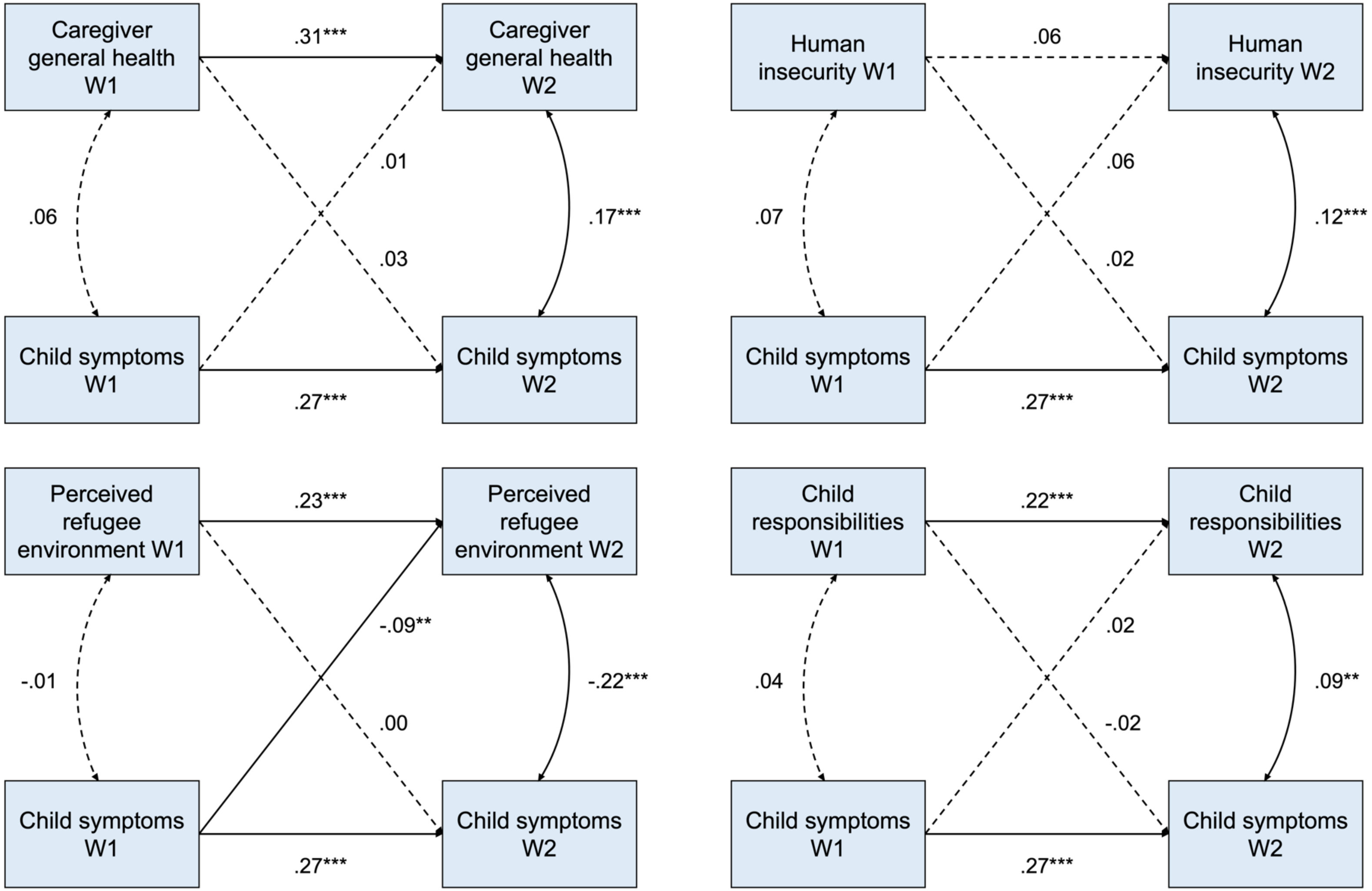

Note. Pathways depict coefficients from 20 individual cross-lagged panel models, for all predictors that emerged as significant in the ANCOVAs. Not shown are pathways controlling for the effects of child age, gender, time since leaving Syria, and war exposure, which were included as predictors for both predictor and child composite symptom score (depression, PTSD, and externalising) at both waves. Dashed pathways are non-significant.* $p < .05$, ** $p < .01$, *** $p < .001$

2.4 Table S3. Analyses of Covariance: Complete Case Results

Factor		Mental health risk trajectory M(SD)				F	Adj. R Squared	Post-hoc comparisons
		Stable High Risk (SHR)	Deteriorating (D)	Improving (I)	Stable Low Risk (SLR)			
Optimism	Baseline	8.93 (2.97)	9.72 (2.51)	9.51 (2.76)	10.06 (2.18)	8.71***	0.41	SHR < I / SLR
	Change	0.37 (4.09)	0.04 (4.03)	0.79 (3.64)	0.46 (2.99)	6.07**	0.40	SHR < I / SLR
Self-efficacy	Baseline	11.13 (3.45)	11.61 (3.19)	11.53 (3.58)	11.22 (3.45)	0.41		
	Change	0.80 (4.54)	0.25 (4.98)	0.41 (4.47)	1.21 (4.22)	0.85		
Self-esteem	Baseline	3.87 (1.26)	4.42 (0.64)	4.08 (1.06)	4.44 (0.70)	9.24***	0.46	SHR < I / SLR
	Change	0.12 (1.40)	-0.33 (1.06)	0.26 (1.15)	-0.07 (1.00)	5.78**	0.41	SHR / D < I
Environmental sensitivity	Baseline	5.14 (1.01)	4.80 (0.95)	5.10 (1.04)	4.94 (0.86)	6.35**	0.50	I / SLR < SHR
	Change	-0.25 (1.30)	0.14 (1.25)	-0.57 (1.24)	-0.30 (1.09)	10.68***	0.49	I / SLR < D<br>I < SHR
Religiosity	Baseline	31.59 (6.36)	32.24 (6.51)	32.07 (6.79)	31.62 (5.77)	0.87		
	Change	2.35 (8.17)	2.27 (8.05)	2.08 (7.93)	2.44 (7.22)	0.17		
Future aspirations	Baseline	3.32 (1.07)	3.47 (0.94)	3.25 (1.08)	3.28 (1.11)	1.58		
	Change	0.04 (1.35)	-0.07 (1.26)	-0.11 (1.44)	-0.04 (1.40)	1.60		
Future expectations	Baseline	2.99 (1.02)	3.06 (0.95)	3.07 (0.97)	2.96 (0.99)	0.60		
	Change	0.11 (1.32)	-0.06 (1.09)	-0.14 (1.33)	0.09 (1.16)	2.40		

Factor		Mental health risk trajectory M(SD)				F	Adj. R Squared	Post-hoc comparisons
		Stable High Risk (SHR)	Deteriorating (D)	Improving (I)	Stable Low Risk (SLR)			
Future planning	Baseline	3.06 (0.86)	3.10 (0.73)	3.03 (0.88)	2.97 (0.92)	2.82		
	Change	0.04 (1.13)	-0.10 (1.11)	-0.26 (1.24)	0.00 (1.16)	6.35**	0.39	I < SHR
Problem-focused coping	Baseline	7.71 (2.62)	7.15 (2.46)	7.85 (2.79)	7.07 (2.51)	1.21		
	Change	0.72 (3.59)	1.36 (3.67)	0.13 (3.74)	0.90 (3.37)	1.63		
Avoidant coping	Baseline	8.05 (2.69)	7.47 (2.48)	8.13 (2.70)	7.11 (2.48)	0.99		
	Change	0.08 (3.63)	0.82 (3.72)	-0.19 (3.88)	1.00 (3.33)	0.73		
Support seeking	Baseline	6.60 (3.08)	5.89 (2.79)	6.23 (2.96)	5.94 (2.90)	1.02		
	Change	0.36 (4.40)	1.18 (4.03)	0.44 (3.91)	1.24 (4.31)	0.64		
Distraction coping	Baseline	6.58 (2.28)	6.56 (2.14)	6.46 (2.29)	6.50 (1.69)	1.96		
	Change	-0.88 (3.11)	-1.25 (2.53)	-0.35 (3.15)	-0.09 (2.45)	5.48**	0.39	SHR / D < SLR<br>D < I
Positive cognitive restructuring	Baseline	8.32 (2.61)	8.47 (2.55)	8.65 (2.65)	7.96 (2.49)	3.27*	0.40	SHR < I
	Change	-0.11 (3.80)	-0.36 (3.49)	0.26 (3.42)	0.72 (3.65)	3.29*	0.40	SHR < I
Child general health	Baseline	2.37 (0.83)	2.16 (0.92)	2.26 (0.78)	2.11 (0.75)	7.80***	0.47	I / SLR < SHR
	Change	-0.30 (1.04)	-0.09 (1.17)	-0.48 (0.90)	-0.33 (0.94)	7.95***	0.47	I / SLR < SHR<br>I < D

Factor		Mental health risk trajectory M(SD)				F	Adj. R Squared	Post-hoc comparisons
		Stable High Risk (SHR)	Deteriorating (D)	Improving (I)	Stable Low Risk (SLR)			
Bullying	Baseline	5.81 (6.91)	3.03 (4.99)	4.16 (5.80)	3.02 (4.68)	10.46***	0.50	D / I / SLR < SHR
	Change	-1.39 (7.88)	0.76 (6.72)	-2.15 (6.78)	-0.89 (5.81)	7.12***	0.45	I < D / SHR
Loneliness and social isolation	Baseline	8.74 (3.00)	7.06 (2.10)	8.29 (2.70)	6.71 (2.21)	24.29***	0.49	SLR < I < SHR<br>D < SHR
	Change	-0.90 (4.10)	-0.20 (3.85)	-1.74 (3.82)	-0.50 (3.06)	12.32***	0.45	I / SLR < SHR
Perceived social support	Baseline	5.53 (0.93)	5.70 (0.74)	5.58 (0.80)	5.73 (0.72)	2.32		
	Change	0.08 (1.29)	0.20 (1.16)	0.18 (1.04)	0.08 (1.03)	2.25		
Maternal acceptance	Baseline	26.96 (4.25)	27.96 (3.07)	27.58 (3.47)	28.33 (2.58)	6.91***	0.30	SHR < I / SLR
	Change	-0.02 (5.24)	-0.13 (4.29)	0.53 (4.35)	0.37 (3.56)	3.35*	0.29	SHR < I
Maternal psychological control	Baseline	11.62 (2.81)	10.53 (1.55)	10.92 (2.11)	10.26 (1.48)	12.91***	0.47	D / I / SLR < SHR
	Change	-0.69 (3.14)	-0.26 (2.01)	-0.67 (2.25)	-0.11 (2.16)	3.56*	0.45	I < SHR
Parent-child conflict	Baseline	6.14 (3.24)	5.87 (3.10)	5.93 (3.27)	5.13 (2.42)	8.32***	0.35	I / SLR < SHR<br>SLR < D
	Change	1.84 (4.93)	1.33 (4.72)	0.58 (4.47)	0.74 (3.69)	9.83***	0.37	I / SLR < SHR
Parental monitoring	Baseline	14.02 (1.98)	14.29 (1.20)	14.13 (1.91)	14.28 (1.86)	1.85		
	Change	-0.06 (2.69)	-0.04 (1.71)	0.29 (2.13)	0.11 (1.84)	2.24		

Factor		Mental health risk trajectory M(SD)				F	Adj. R Squared	Post-hoc comparisons
		Stable High Risk (SHR)	Deteriorating (D)	Improving (I)	Stable Low Risk (SLR)			
Positive home experiences	Baseline	15.76 (3.93)	15.02 (3.89)	15.99 (4.02)	15.88 (3.49)	0.35		
	Change	-0.34 (5.42)	1.33 (5.30)	-0.52 (5.30)	0.12 (4.51)	1.19		
Child maltreatment	Baseline	13.65 (12.89)	7.45 (7.51)	11.16 (12.01)	7.47 (8.37)	20.74***	0.49	D / I / SLR < SHR
	Change	-2.55 (14.78)	1.92 (10.33)	-6.57 (12.27)	-3.24 (10.48)	21.10***	0.48	I / SLR < D / SHR
Caregiver depression	Baseline	15.96 (6.42)	12.60 (6.38)	14.90 (6.13)	12.89 (6.34)	21.24***	0.34	D / I / SLR < SHR
	Change	-0.09 (7.81)	1.31 (7.75)	-4.14 (8.39)	-3.89 (8.13)	33.33***	0.34	I / SLR < D / SHR
Caregiver PTSD	Baseline	35.40 (17.20)	28.12 (14.22)	33.56 (17.33)	28.25 (18.18)	14.58***	0.46	I / SLR < SHR
	Change	-7.56 (22.42)	-2.65 (20.47)	-16.60 (24.88)	-12.61 (21.47)	21.81***	0.44	I / SLR < D / SHR
Caregiver anxiety	Baseline	8.60 (5.36)	6.18 (4.84)	8.14 (5.30)	6.90 (5.23)	10.29***	0.43	D / I / SLR < SHR
	Change	-0.67 (6.59)	0.29 (5.52)	-2.88 (6.41)	-2.00 (6.97)	14.05***	0.41	I / SLR < SHR<br>I < D
Caregiver general health	Baseline	3.04 (0.90)	2.88 (0.99)	2.97 (0.93)	2.74 (0.94)	4.21*	0.38	I < SHR
	Change	-0.18 (1.06)	-0.08 (1.06)	-0.43 (1.08)	-0.06 (1.14)	5.72**	0.39	I < SHR
Human insecurity	Baseline	3.70 (0.37)	3.64 (0.41)	3.66 (0.44)	3.70 (0.43)	5.40**	0.51	I < SHR
	Change	0.07 (0.47)	0.16 (0.53)	-0.02 (0.63)	-0.06 (0.61)	7.82***	0.51	I / SLR < D / SHR
Perceived refugee	Baseline	3.22 (0.52)	3.26 (0.53)	3.29 (0.50)	3.13 (0.51)	9.22***	0.47	SHR / D < I

Factor		Mental health risk trajectory M(SD)				F	Adj. R Squared	Post-hoc comparisons
		Stable High Risk (SHR)	Deteriorating (D)	Improving (I)	Stable Low Risk (SLR)			
environment	Change	0.01 (0.60)	0.06 (0.59)	0.20 (0.61)	0.31 (0.63)	15.14***	0.43	SHR / D < I<br>SHR < SLR
Collective efficacy	Baseline	32.03 (6.12)	32.43 (5.92)	32.55 (6.38)	32.59 (6.92)	0.94		
	Change	-0.90 (9.17)	-0.30 (10.19)	-0.11 (9.38)	-0.91 (10.98)	1.00		
Household size	Baseline	7.51 (2.45)	7.58 (2.38)	7.88 (2.45)	7.76 (2.53)	1.47		
	Change	-0.03 (2.07)	0.13 (1.99)	0.12 (2.29)	0.00 (2.03)	0.84		
School attendance	Baseline	1.12 (0.85)	1.04 (0.90)	1.04 (0.88)	0.87 (0.86)	3.38*	0.40	
	Change	-0.35 (0.86)	-0.29 (0.91)	-0.44 (0.83)	-0.18 (0.94)	2.20		
Parent abroad	Baseline	0.14 (0.35)	0.06 (0.23)	0.15 (0.35)	0.09 (0.28)	1.13		
	Change	-0.03 (0.36)	0.03 (0.32)	-0.01 (0.39)	0.00 (0.27)	0.41		
Child responsibilities	Baseline	4.41 (3.45)	3.60 (2.82)	4.26 (3.28)	4.18 (2.70)	1.83		
	Change	1.15 (4.06)	1.73 (3.83)	0.43 (3.93)	1.22 (3.95)	4.58**	0.35	I < D / SHR
Parent deceased	Baseline	0.09 (0.28)	0.07 (0.25)	0.08 (0.26)	0.05 (0.22)	0.24		
	Change	0.01 (0.21)	-0.01 (0.18)	0.00 (0.14)	0.01 (0.11)	0.37		
Caregiver employed	Baseline	1.88 (0.33)	1.89 (0.32)	1.88 (0.33)	1.93 (0.26)	0.77		
	Change	-0.08 (0.45)	-0.06 (0.41)	-0.03 (0.45)	-0.07 (0.47)	1.10		

Factor		Mental health risk trajectory M(SD)				F	Adj. R Squared	Post-hoc comparisons
		Stable High Risk (SHR)	Deteriorating (D)	Improving (I)	Stable Low Risk (SLR)			
Family income	Baseline	1.87 (1.07)	1.94 (1.07)	1.92 (1.09)	1.84 (1.07)	0.09		
	Change	0.09 (1.50)	0.09 (1.25)	0.06 (1.57)	0.21 (1.27)	0.13		
Adult literacy	Baseline	2.62 (1.17)	2.60 (1.16)	2.52 (1.15)	2.48 (1.19)	0.82		
	Change	0.05 (1.40)	-0.22 (1.36)	0.03 (1.31)	0.02 (1.53)	1.57		

Note. Table representing analyses of covariance from all predictors using complete case data (N = 861). Child age, gender, time since leaving Syria, and war exposure were entered as covariates into all ANCOVAs. Baseline models controlled for change scores, and change models controlled for baseline scores. F statistic is based on test against null model including only covariates. Adjusted R Square is based on full model. Post-hoc comparisons are based on Tukey's test. Means and SDs are unadjusted estimates, all other statistics are based on adjusted means according to the ANCOVA models. Higher scores on child and caregiver general health indicate worse health. Post-hoc comparisons for the change scores depict relationships based on the actual change score (differentiating change in negative and positive directions), not relative amount of change. P-values based on Benjamini-Hochberg correction for multiple testing (Benjamini & Hochberg 1995) * p < .05, ** p < .01, *** p < .00

2.5 Figure S3. Cross-Lagged Panel Models: Complete case data

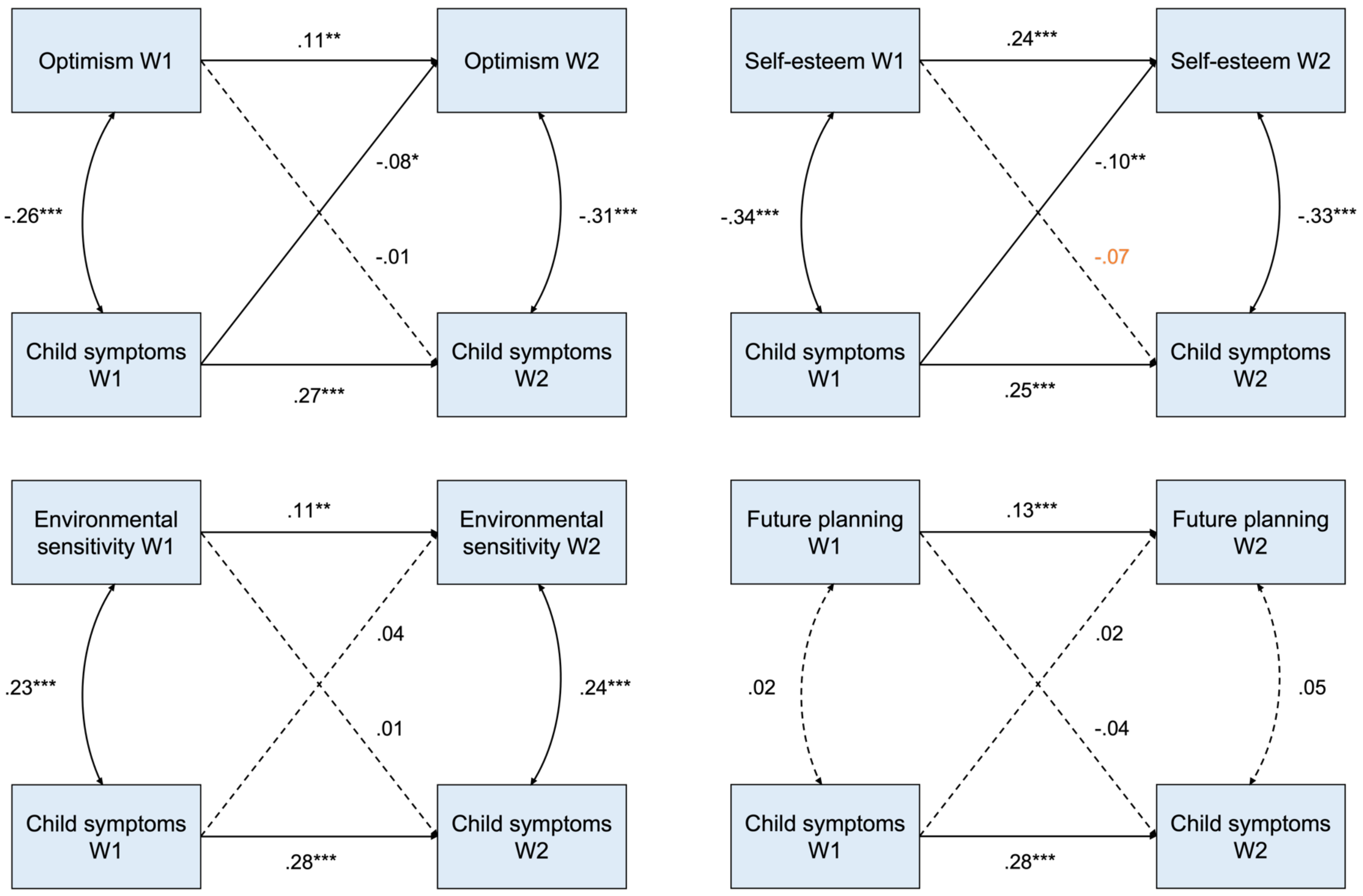

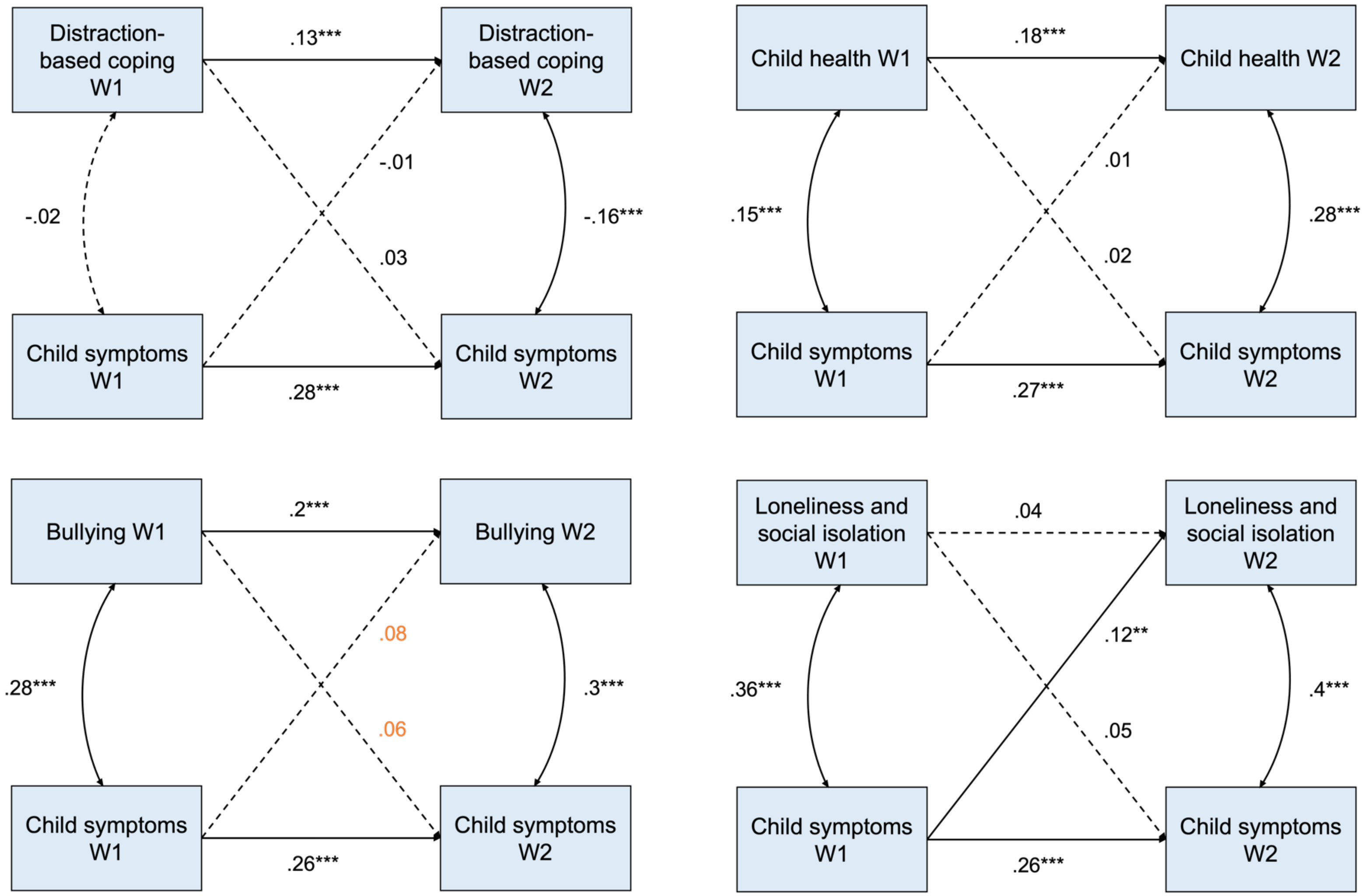
Distraction-based coping W1
Distraction-based coping W2
Child symptoms W1
Child symptoms W2
.13***
-.01
.03
-.02
-.16***
.28***
Child health W1
Child health W2
Child symptoms W1
Child symptoms W2
.18***
.01
.02
.15***
.28***
.27***
Bullying W1
Bullying W2
Child symptoms W1
Child symptoms W2
.2***
.08
.06
.28***
.3***
.26***
Loneliness and social isolation W1
Loneliness and social isolation W2
Child symptoms W1
Child symptoms W2
.04
.12**
.05
.36***
.4***
.26***

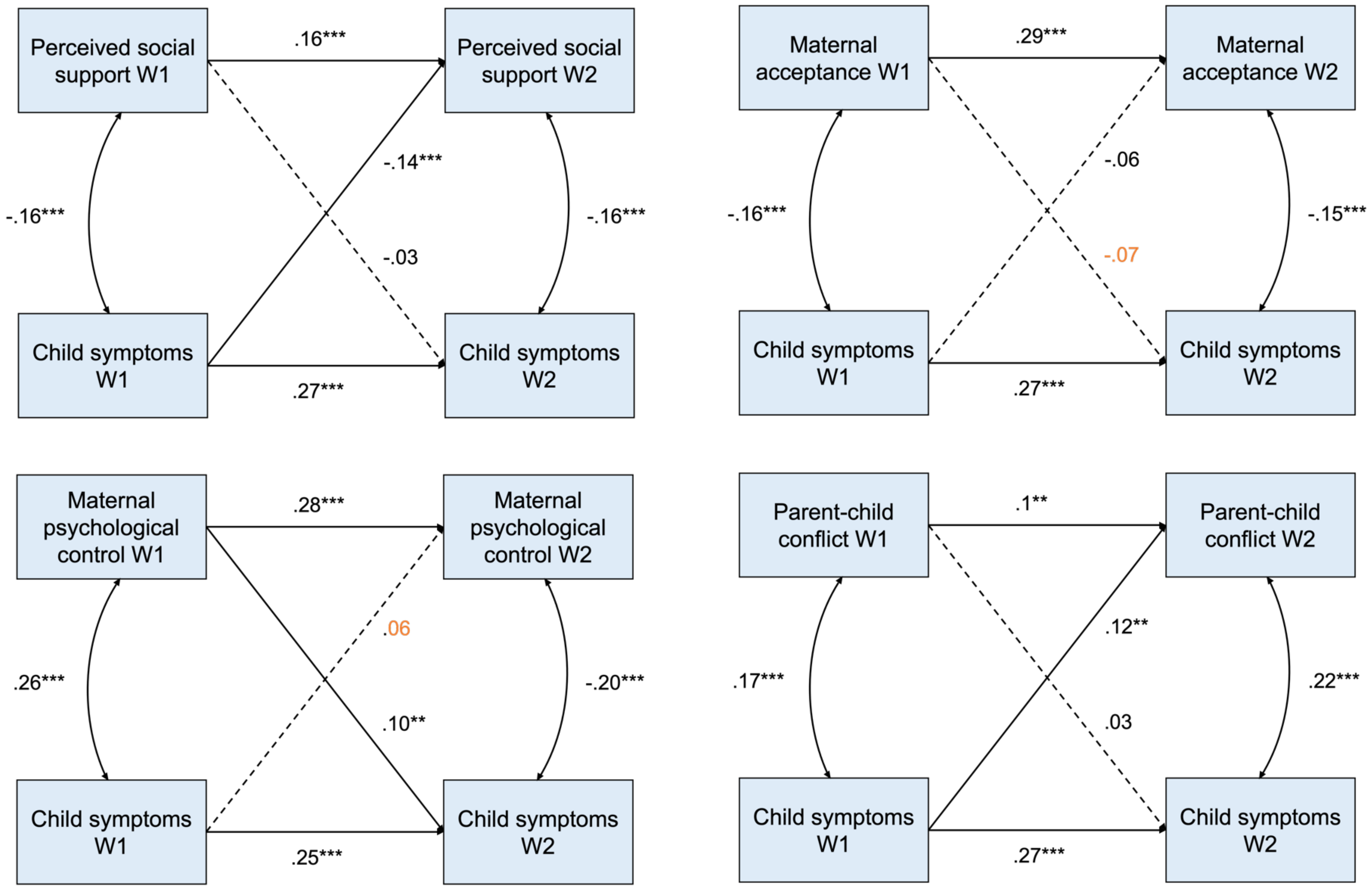
Perceived social support W1
Perceived social support W2
Child symptoms W1
Child symptoms W2
.16***
-.16***
-.14***
-.03
-.16***
.27***
Maternal acceptance W1
Maternal acceptance W2
Child symptoms W1
Child symptoms W2
.29***
-.16***
-.06
-.07
-.15***
.27***
Maternal psychological control W1
Maternal psychological control W2
Child symptoms W1
Child symptoms W2
.28***
.26***
.06
.10**
-.20***
.25***
Parent-child conflict W1
Parent-child conflict W2
Child symptoms W1
Child symptoms W2
.1**
.17***
.12**
.03
.22***
.27***

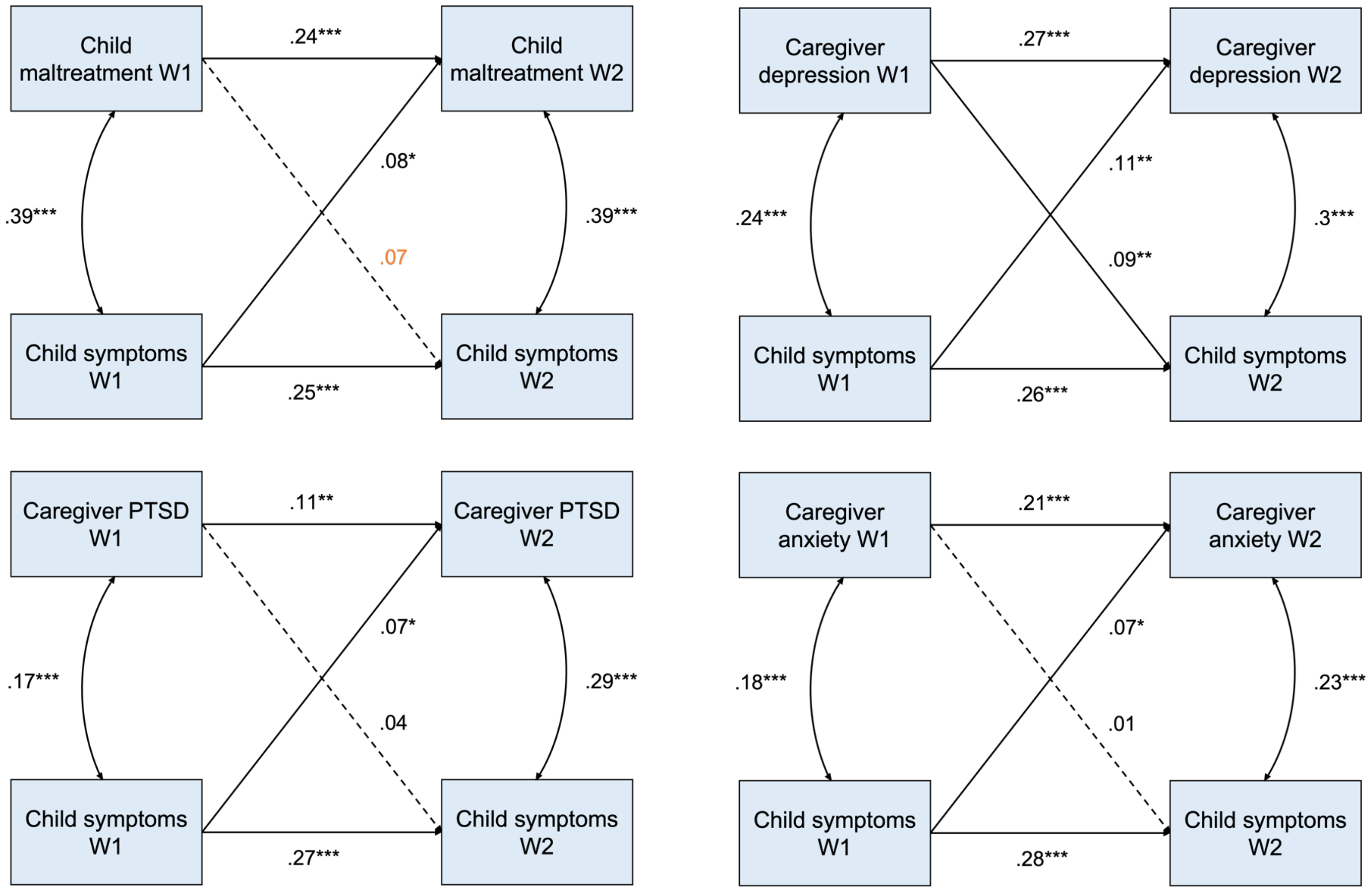
Child maltreatment W1
Child maltreatment W2
Child symptoms W1
Child symptoms W2
.24***
.08*
.07
.39***
.39***
.25***
Caregiver depression W1
Caregiver depression W2
Child symptoms W1
Child symptoms W2
.27***
.11**
.09**
.24***
.3***
.26***
Caregiver PTSD W1
Caregiver PTSD W2
Child symptoms W1
Child symptoms W2
.11**
.07*
.04
.17***
.29***
.27***
Caregiver anxiety W1
Caregiver anxiety W2
Child symptoms W1
Child symptoms W2
.21***
.07*
.01
.18***
.23***
.28***

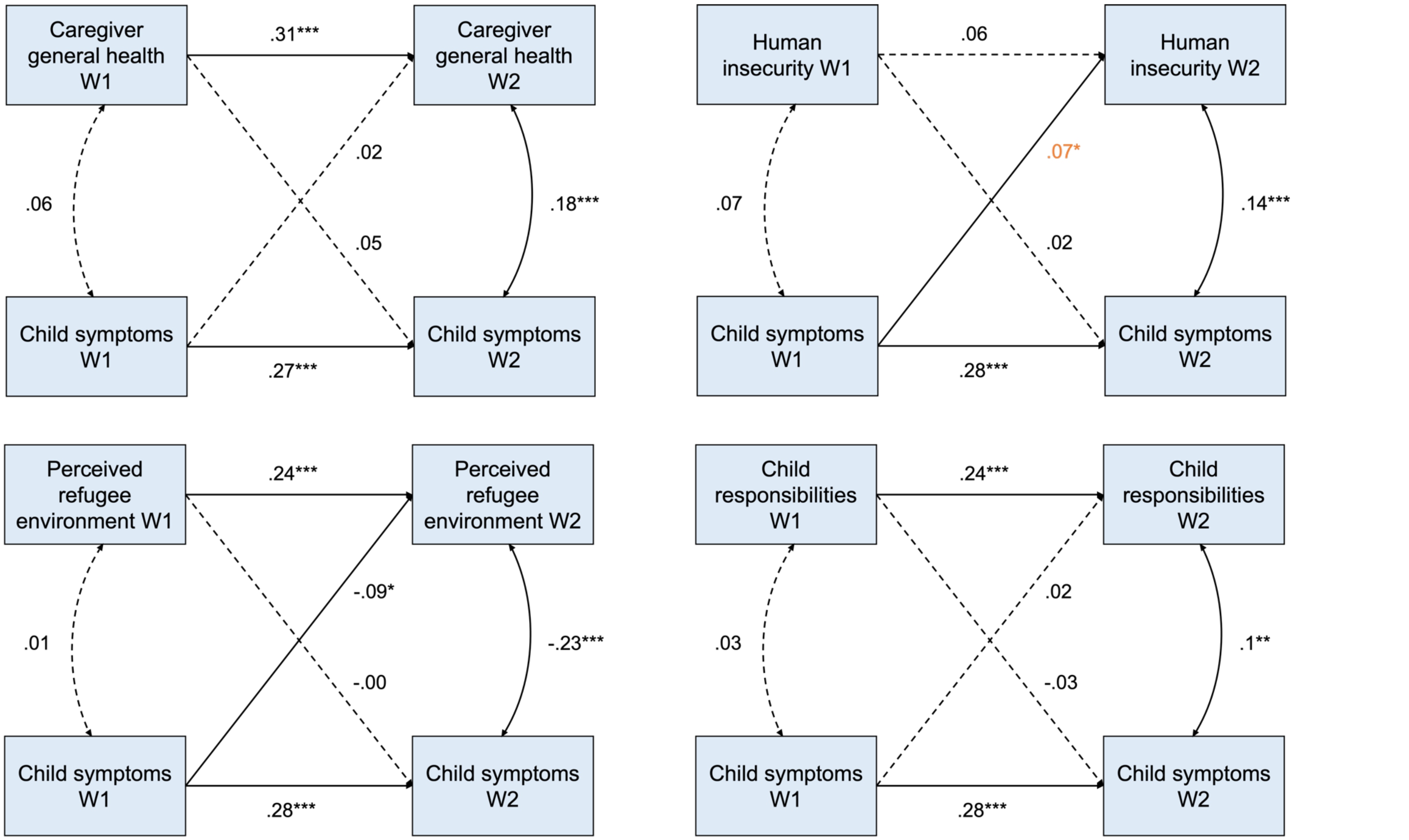

Note. Pathways depict coefficients from cross-lagged panel models. Not shown are pathways controlling for the effects of child age, gender, time since leaving Syria, and war exposure, which were included as predictors for both predictor and child composite symptom score (depression, PTSD, and externalising) at both waves. Dashed pathways are non-significant. Coefficients in orange are pathways that were significant in the imputed data but not in this complete case analysis, or vice versa.* $p < .05$, ** $p < .01$, *** $p < .001$

3. References

Abdo, H. A. (2016). Depressive Symptoms among Adolescents in Lebanon: a Confirmatory Factor Analytic Study of the Center for Epidemiological Studies Depression for Children. *Acta Psychopathologica*, **02**(06). doi:10.4172/2469-6676.100072

Alyahri, A., & Goodman, R. (2006). Validation of the Arabic Strengths and Difficulties Questionnaire and the Development and Well-Being Assessment. *Undefined*.

Arakelyan, S., & Ager, A. (2021). Annual Research Review: A multilevel bioecological analysis of factors influencing the mental health and psychosocial well-being of refugee children. *Journal of Child Psychology and Psychiatry*, **62**(5), 484–509.

Asher, S. R., Hymel, S., & Renshaw, P. D. (1984). Loneliness in Children. *Child Development*, **55**(4), 1456.

Barber, B. K. (1996). Parental Psychological Control: Revisiting a Neglected Construct. *Child Development*, **67**(6), 3296.

Barber, B. K. (1999). Political Violence, Family Relations, and Palestinian Youth Functioning. *Journal of Adolescent Research*, **14**(2), 206–230.

Barber, B. K. (2001). Political violence, social integration, and youth functioning: Palestinian youth from the Intifada. *Journal of Community Psychology*, **29**(3), 259–280.

Barber, B. K., Xia, M., Olsen, J. A., McNeely, C. A., & Bose, K. (2012). Feeling disrespected by parents: Refining the measurement and understanding of psychological control. *Journal of Adolescence*, **35**(2), 273–287.

Bech, P. (2012). Clinical Psychometrics. *Clinical Psychometrics*. doi:10.1002/9781118511800

Benjamini, Y., & Hochberg, Y. (1995). Controlling the False Discovery Rate: A Practical and Powerful Approach to Multiple Testing. *Journal of the Royal Statistical Society: Series B (Methodological)*, **57**(1), 289–300.

Berthold, S. M., Kong, S., Mollica, R. F., Kuoch, T., Scully, M., & Franke, T. (2014). Comorbid Mental and Physical Health and Health Access in Cambodian Refugees in the US. *Journal of Community Health*, **39**(6), 1045–1052.

Betancourt, T. S., Salhi, C., Buka, S., Leaning, J., Dunn, G., & Earls, F. (2012). Connectedness, social support and internalising emotional and behavioural problems in adolescents displaced by the Chechen conflict. *Disasters*, **36**(4), 635–655.

Blevins, C. A., Weathers, F. W., Davis, M. T., Witte, T. K., & Domino, J. L. (2015). The Posttraumatic Stress Disorder Checklist for DSM-5 (PCL-5): Development and Initial Psychometric Evaluation. *Journal of Traumatic*

Stress, **28**(6), 489–498.

Brent Donnellan, M., Kenny, D. A., Trzesniewski, K. H., Lucas, R. E., & Conger, R. D. (2012). Using trait–state models to evaluate the longitudinal consistency of global self-esteem from adolescence to adulthood. *Journal of Research in Personality*, **46**(6), 634–645.

Brown, K., & Westaway, E. (2011). Agency, Capacity, and Resilience to Environmental Change: Lessons from Human Development, Well-Being, and Disasters. *Https://Doi.Org/10.1146/Annurev-Environ-052610-092905*, **36**, 321–342.

Çeri, V., Ak, F., Fındık, O. T. P., Arman, A., Fiş, N.P., Beser, C., Göksu, M., & Fazel, M. (2021). Syrian refugee children face more peer victimization in schools what leads to poor mental health: a brief report. *European Child & Adolescent Psychiatry*, **30**(9), 1475–1477.

Chen, W., Hall, B. J., Ling, L., & Renzaho, A. M. (2017). Pre-migration and post-migration factors associated with mental health in humanitarian migrants in Australia and the moderation effect of post-migration stressors: findings from the first wave data of the BNLA cohort study. *The Lancet. Psychiatry*, **4**(3), 218–229.

Chung, M. C., AlQarni, N., Al Muhairi, S., & Mitchell, B. (2017). The relationship between trauma centrality, self-efficacy, posttraumatic stress and psychiatric co-morbidity among Syrian refugees: Is gender a moderator? *Journal of Psychiatric Research*, **94**, 107–115.

Elklit, A., Østergård Kjær, K., Lasgaard, M., & Palic, S. (2012). Social support, coping and posttraumatic stress symptoms in young refugees. *Torture : Quarterly Journal on Rehabilitation of Torture Victims and Prevention of Torture*, **22**(1), 11–23.

Eltanamly, H., Leijten, P., Jak, S., & Overbeek, G. (2021). Parenting in Times of War: A Meta-Analysis and Qualitative Synthesis of War Exposure, Parenting, and Child Adjustment. *Trauma, Violence, and Abuse*, **22**(1), 147–160.

Eruyar, S., Maltby, J., & Vostanis, P. (2018). Mental health problems of Syrian refugee children: the role of parental factors. *European Child & Adolescent Psychiatry*, **27**(4), 401–409.

Ey, S., Hadley, W., Allen, D. N., Palmer, S., Klosky, J., Deptula, D., Thomas, J., & Cohen, R. (2005). A new measure of children's optimism and pessimism: The youth life orientation test. *Journal of Child Psychology and Psychiatry and Allied Disciplines*, **46**(5), 548–558.

Faulstich, M. E., Carey, M. P., Ruggiero, L., Enyart, P., & Gresham, F. (1986). Assessment of depression in childhood and adolescence: an evaluation of the Center for Epidemiological Studies Depression Scale for Children (CES-DC). *American Journal of Psychiatry*, **143**(8), 1024–1027.

Foa, E. B., Johnson, K. M., Feeny, N. C., & Treadwell, K. R. H. (2001). The Child PTSD Symptom Scale: A Preliminary Examination of its Psychometric Properties. *Journal of Clinical Child & Adolescent Psychology*, **30**(3), 376–384.

Goodman, A., Lamping, D. L., & Ploubidis, G. B. (2010). When to use broader internalising and externalising subscales instead of the hypothesised five subscales on the strengths and difficulties questionnaire (SDQ): Data from british parents, teachers and children. *Journal of Abnormal Child Psychology*, **38**(8), 1179–1191.

Goodman, R. (1997). The Strengths and Difficulties Questionnaire: a research note. *Journal of Child Psychology and Psychiatry, and Allied Disciplines*, **38**(5), 581–6.

Habib, R. R., Ziadee, M., Abi Younes, E., Harastani, H., Hamdar, L., Jawad, M., & El Asmar, K. (2019). Displacement, deprivation and hard work among Syrian refugee children in Lebanon. *BMJ Global Health*, **4**(1). doi:10.1136/BMJGH-2018-001122

Harris, M. A., Donnellan, M. B., & Trzesniewski, K. H. (2018). The Lifespan Self-Esteem Scale: Initial Validation of a New Measure of Global Self-Esteem. *Journal of Personality Assessment*, **100**(1), 84–95.

Henry, J. D., & Crawford, J. R. (2005). The short-form version of the Depression anxiety stress scales (DASS-21): Construct validity and normative data in a large non-clinical sample. *British Journal of Clinical Psychology*, **44**(2), 227–239.

Javanbakht, A., Rosenberg, D., Haddad, L., & Arfken, C. L. (2018). Mental Health in Syrian Refugee Children Resettling in the United States: War Trauma, Migration, and the Role of Parental Stress. *Journal of the American Academy of Child and Adolescent Psychiatry*, **57**(3), 209-211.e2.

Johnson, S. R. L., Blum, R. W., & Cheng, T. L. (2014). Future orientation: A construct with implications for adolescent health and wellbeing. *International Journal of Adolescent Medicine and Health*, Walter de Gruyter GmbH, pp. 459–468.

Karam, E. G., Al-Atrash, R., Saliba, S., Melhem, N., & Howard, D. (1999). The War Events Questionnaire. *Social Psychiatry and Psychiatric Epidemiology*, **34**(5), 265–274.

Karam, E. G., Fayyad, J. A., Farhat, C., Pluess, M., Haddad, Y.C., Tabet, C.C., Farah, L., & Kessler, R. C. (2019). Role of childhood adversities and environmental sensitivity in the development of post-traumatic stress disorder in war-exposed Syrian refugee children and adolescents. *The British Journal of Psychiatry*, 1–7.

Kessler, R. C., & Ustün, T. B. (2004). The World Mental Health (WMH) Survey Initiative Version of the World Health Organization (WHO) Composite International Diagnostic Interview (CIDI). *International Journal of Methods in Psychiatric Research*, **13**(2), 93–117.

Lau, W., Silove, D., Edwards, B., Forbes, D., Bryant, R., McFarlane, A., Hadzi-Pavlovic, D., Steel, Z., Nickerson, A., Van Hooff, M., Felmingham, K., Cowlishaw, S., Alkemade, N., Kartal, D., & O'Donnell, M. (2018). Adjustment of refugee children and adolescents in Australia: Outcomes from wave three of the Building a New Life in Australia study. *BMC Medicine*, **16**(1), 1–17.

Marley, C., & Mauki, B. (2019). Resilience and protective factors among refugee children post-migration to high-income countries: a systematic review. *European Journal of Public Health*, **29**(4), 706–713.

McEwen, F. S., Moghames, P., Bosqui, T. J., Kyrillos, V., Chehade, N., Saad, S., Abdul Rahman, D., Popham, C.M., Saab, D., Karam, G., Karam, E.G., & Pluess, M. (2020). Validating screening questionnaires for internalizing and externalizing disorders against clinical interviews in 8-17 year-old Syrian refugee children. *Technical working paper*. Retrieved from https://psyarxiv.com/6zu87/

McEwen, F. S., Popham, C. M., Moghames, P., Smeeth, D., De Villiers, B., Saab, D., Karam, G., Fayyad, J., Karam, E., & Pluess, M. (2022). Cohort profile: biological pathways of risk and resilience in Syrian refugee children (BIOPATH). *Social Psychiatry and Psychiatric Epidemiology 2022*, **1**, 1–11.

McGregor, L. S., Melvin, G. A., & Newman, L. K. (2015). Familial separations, coping styles, and PTSD symptomatology in resettled refugee youth. *Journal of Nervous and Mental Disease*, **203**(6), 431–438.

Meyer, S. R., Yu, G., Rieders, E., & Stark, L. (2020). Child labor, sex and mental health outcomes amongst adolescent refugees. *Journal of Adolescence*, **81**, 52–60.

Nagi, Y., Sender, H., Orcutt, M., Fouad, F., Burgess, R. A., & Devakumar, D. (2021). Resilience as a communal concept: Understanding adolescent resilience in the context of the Syrian refugee crisis in Bar Elias, Lebanon. *Journal of Migration and Health*, **3**, 100046.

Nasıroğlu, S., Çeri, V., Erkorkmaz, Ü., & Semerci, B. (2018). Determinants of psychiatric disorders in children refugees in Turkey's Yazidi refugee camp. *Psychiatry and Clinical Psychopharmacology*, **28**(3), 291–299.

Nyarko, F., & Punamaki, R. L. (2020). Future orientation of youth with a history of war trauma: a qualitative study in the African context. *Https://Doi.Org/10.1080/13623699.2019.1706879*, **35**(4), 313–335.

Pearce, L. D., Foster, E. M., & Hardie, J. H. (2013). A person-centered examination of adolescent religiosity using latent class analysis. *Journal for the Scientific Study of Religion*, **52**(1), 57–79.

Pluess, M. (2015). Individual Differences in Environmental Sensitivity. *Child Development Perspectives*, **9**(3), 138–143.

Pluess, M., Assary, E., Lionetti, F., Lester, K.J., Krapohl, E., Aran, E.N., & Aron, A. (2018). Environmental sensitivity in children: Development of the Highly Sensitive Child Scale and identification of sensitivity groups.

Developmental Psychology, **54**(1), 51–70.

Program for Prevention Research. (1999). *Manual for the Children's Coping Strategies Checklist and the How I Coped Under Pressure Scale*, Tempe, AZ.

Radloff, L. S. (1977). The CES-D Scale. *Applied Psychological Measurement*, **1**(3), 385–401.

Ramaswamy, V., Aroian, K. J., & Templin, T. (2009). Adaptation and psychometric evaluation of the multidimensional scale of perceived social support for arab American adolescents. *American Journal of Community Psychology*, **43**(1–2), 49–56.

Runyan, D. K., Dunne, M. P., & Zolotor, A. J. (2009). Introduction to the development of the ISPCAN child abuse screening tools. *Child Abuse and Neglect*, **33**(11), 842–845.

Samara, M., El Asam, A., Khadaroo, A., & Hammuda, S. (2020). Examining the psychological well-being of refugee children and the role of friendship and bullying. *British Journal of Educational Psychology*, **90**(2), 301–329.

Sampson, R. J., Raudenbush, S. W., & Earls, F. (1997). Neighborhoods and violent crime: A multilevel study of collective efficacy. *Science*, **277**(5328), 918–924.

Sapmaz, Ş. Y., Tanrıverdi, B. U., Öztürk, M., Gözaçanlar, Ö., Ülker, G. Y., & Özkan, Y. (2017). Immigration-related mental health disorders in refugees 5–18 years old living in Turkey. *Neuropsychiatric Disease and Treatment*, **13**, 2813–2821.

Schaefer, E. S. (1965). Children's Reports of Parental Behavior: An Inventory. *Child Development*, **36**(2), 413.

Scharpf, F., Kaltenbach, E., Nickerson, A., & Hecker, T. (2021). A systematic review of socio-ecological factors contributing to risk and protection of the mental health of refugee children and adolescents. *Clinical Psychology Review*, Elsevier Inc., p. 101930.

Scherer, N., Hameed, S., Acarturk, C., Deniz, G., Sheikhani, A., Volkan, S., Örücü, A., Pivato, I., Akıncı, İ., Patterson, A., & Polack, S. (2020). Prevalence of common mental disorders among Syrian refugee children and adolescents in Sultanbeyli district, Istanbul: Results of a population-based survey. *Epidemiology and Psychiatric Sciences*, **29**. doi:10.1017/S2045796020001079

Schwarzer, R., & Jerusalem, M. (1995). Generalized Self-Efficacy scale. In J. Weinman, S. Wright, & M. Johnston, eds., *Measures in health psychology: A user's portfolio. Causal and control beliefs*, Windsor, England: NFER-NELSON, pp. 35–37.

Sibai, A. M., Chaaya, M., Tohme, R. A., Mahfoud, Z., & Al-Amin, H. (2009). Validation of the Arabic version of the 5-item WHO well being index in elderly population. *International Journal of Geriatric Psychiatry*, **24**(1),

106–107.

Sleijpen, M., Haagen, J., Mooren, T., & Kleber, R. J. (2016). Growing from experience: An exploratory study of posttraumatic growth in adolescent refugees. *European Journal of Psychotraumatology*, **7**(1), 28698.

Speidel, R., Galarneau, E., Elsayed, D., Mahhouk, S., Filippelli, J., Colasante, T., & Malti, T. (2021). Refugee children's social–emotional capacities: Links to mental health upon resettlement and buffering effects on pre-migratory adversity. *International Journal of Environmental Research and Public Health*, **18**(22). doi:10.3390/IJERPH182212180

Strathman, A., Gleicher, F., Boninger, D. S., & Edwards, C. S. (1994). The Consideration of Future Consequences: Weighing Immediate and Distant Outcomes of Behavior. *Journal of Personality and Social Psychology*, **66**(4), 742–752.

Suldo, S. M., & Fefer, S. A. (2013). Parent-Child Relationships and Well-Being. *Research, Applications, and Interventions for Children and Adolescents: A Positive Psychology Perspective*, 131–147.

Tol, W. A., Song, S., & Jordans, M. J. D. (2013). Annual Research Review: Resilience and mental health in children and adolescents living in areas of armed conflict - a systematic review of findings in low- and middle-income countries. *Journal of Child Psychology and Psychiatry*, **54**(4), 445–460.

Topp, C. W., Østergaard, S. D., Søndergaard, S., & Bech, P. (2015). The WHO-5 well-being index: A systematic review of the literature. *Psychotherapy and Psychosomatics*, **84**(3), 167–176.

Van Heemstra, H. E., Scholte, W. F., Nickerson, A., Boelen, P.A., Mwanri, L., Gesesew, H., Fauk, N.K., & Mude, W. (2021). Can Circumstances Be Softened? Self-Efficacy, Post-Migratory Stressors, and Mental Health among Refugees. *International Journal of Environmental Research and Public Health*, **18**(4), 1–9.

Weissman, M. M., Orvaschel, H., & Padian, N. (1980). Children's symptom and social functioning self-report scales comparison of mothers' and children's reports. *Journal of Nervous and Mental Disease*, **168**(12), 736–740.

Wiegersma, P. A., Stellinga-Boelen, A. A. M., & Reijneveld, S. A. (2011). Psychosocial Problems in Asylum Seekers' Children. *The Journal of Nervous and Mental Disease*, **199**(2), 85–90.

Zevulun, D., Post, W. J., Zijlstra, A. E., Kalverboer, M. E., & Knorth, E. J. (2017). Migrant and asylum-seeker children returned to Kosovo and Albania: predictive factors for social–emotional wellbeing after return. *Https://Doi.Org/10.1080/1369183X.2017.1391076*, **44**(11), 1774–1796.

Zhang, J., Zhao, G., Li, X., Hong, Y., Fang, X., Barnett, D., Lin, X., Zhao, J., & Zhang, L. (2009). Positive future orientation as a mediator between traumatic events and mental health among children affected by HIV/AIDS in rural China. *AIDS Care - Psychological and Socio-Medical Aspects of AIDS/HIV*, **21**(12), 1508–

1516.

Ziadni, M., Hammoudeh, W., Abu Rmeileh, N. M. E., Hogan, D., Shannon, H., & Giacaman, R. (2011). Sources of human insecurity in post-war situations: The case of Gaza. *Journal of Human Security*, **7**(3), 23–36.
